# Education shares distinct genetic influences with substance use and disorder

**DOI:** 10.1017/S0033291726103353

**Published:** 2026-02-02

**Authors:** Christal N. Davis, Yousef Khan, Zachary Piserchia, Joshua C. Gray, Henry R. Kranzler

**Affiliations:** 1 Perelman School of Medicine, University of Pennsylvania, USA; 2 Crescenz VA Medical Center, USA; 3 Uniformed Services University: Uniformed Services University of the Health Sciences, USA

**Keywords:** alcohol use, alcohol use disorder, cannabis use, cannabis use disorder, cognitive traits, educational attainment, genetic pleiotropy, non-cognitive traits, polygenic overlap, substance use

## Abstract

**Background:**

Educational attainment (EA), which comprises cognitive (CogEA) and noncognitive (NonCogEA) components, is positively genetically correlated with alcohol and cannabis use but negatively correlated with alcohol and cannabis use disorders (AUD and CUD). These paradoxical associations suggest that shared genetic influences with EA may differ by level of substance involvement.

**Methods:**

To test this, we examined the shared genetic architecture of EA, CogEA, and NonCogEA with alcohol consumption (AC), AUD, lifetime cannabis use (CanUse), and CUD. We used bivariate causal mixture models, local genetic correlation analyses, and conditional/conjunctional false discovery rate analyses to identify global, regional, and variant-level overlap for EA and substance-related trait pairs.

**Results:**

EA shared 57.57% of causal variants with AC and 62.42% with AUD, while sharing 48.07% of causal variants with CanUse and 84.18% with CUD. Among shared variants for AC, 48.12% had concordant effects with CogEA and 52.86% with NonCogEA. For AUD, 38.40% and 41.02% of causal variants had concordant effects with CogEA and NonCogEA, respectively. CanUse had higher concordance with CogEA (71.42%) and NonCogEA (65.56%) than CUD (37.97% and 42.23%, respectively). Functional enrichment in brain tissues varied across substance use and EA pairs.

**Conclusions:**

EA is associated with greater alcohol and cannabis use and lower risk for AUD and CUD, a pattern that reflects both concordant and discordant variant effects. CogEA and NonCogEA show partially distinct patterns, particularly for cannabis-related traits, highlighting the importance of disaggregating EA to clarify the genetic architecture underlying its paradoxical associations with substance-related traits.

## Introduction

Educational attainment (EA) is a complex, multifaceted trait influenced by cognitive abilities (e.g. intelligence) and noncognitive attributes (e.g. perseverance and personality) (Demange et al., 2021). In many countries, including the United States, EA significantly influences socioeconomic status and physical and mental health (Demange, Boomsma, van Bergen, & Nivard, 2024; Kondirolli & Sunder, 2022; LaVeist et al., 2023). Given these far-reaching implications, a clearer understanding of how the genetic architecture of EA intersects with that of other complex traits, including substance use and substance use disorders (SUDs), could provide insight into the biological and behavioral pathways that link education to health.

Genetic associations between EA and substance use behaviors are at times paradoxical. Although EA is positively genetically correlated with alcohol and cannabis use (Kranzler et al., 2019; Pasman et al., 2018), it has an equally negative genetic correlation (*r*_g_) with alcohol use disorder (AUD) and cannabis use disorder (CUD) (Kranzler et al., 2019; Levey et al., 2023). One explanation for these findings is that genetic influences on EA promote experimentation or regular low-level substance use without increasing SUD risk. For example, genetic variants associated with openness and curiosity – both important for academic success (Chen, Cheung, & Zeng, 2025) – may encourage experimentation with substances (Moncel, Osmont, & Dauvier, 2025), while other etiologic factors shape the transition to disorder. Similarly, higher socioeconomic status may increase individuals’ access to and opportunities for substance use while providing them with social and material resources that can buffer against the harmful consequences of use (Probst et al., 2020). A more complete understanding of these genetic associations requires moving beyond broad *r*_g_ to examine the contributions of specific genomic regions and individual variants to the patterns observed.

To this end, we conducted analyses that evaluate shared genetic architecture across three increasingly specific levels of resolution: global genome-wide overlap, local regional covariance, and single-nucleotide polymorphism (SNP)-level cross-trait enrichment. First, we assessed the global polygenic overlap for each trait pair – EA/alcohol consumption (AC), EA/CanUse, EA/AUD, and EA/CUD – using bivariate causal mixture models, which estimate the total proportion of shared causal variants between two traits irrespective of their direction of effect (Frei et al., 2019; Holland et al., 2020). MiXeR quantifies *how much* of the genetic architecture is shared, but it does not identify *where* in the genome those shared effects arise. Next, we conducted Local Analysis of [co]Variant Associations (LAVAs) (Werme, van der Sluis, Posthuma, & de Leeuw, 2022) to identify genomic regions where EA shows significant *r*_g_ with each substance-related trait. We then performed conditional and conjunctional false discovery rate (cond/conjFDR) analyses (Andreassen et al., 2013) to identify individual genetic variants that contribute to the overlap between EA and substance use traits, including in those regions without significant local *r*_g_. Comparing the findings across traits and methods enabled us to identify patterns that differentiate the association of EA with substance use from its association with SUDs.

To refine our understanding, we performed similar analyses focusing on the cognitive (CogEA) and noncognitive (NonCogEA) components of EA (Demange et al., 2021). Although a published genome-wide association study (GWAS) showed no significant differences between CogEA and NonCogEA in their *r*_g_ with alcohol dependence, alcohol use, and cannabis use (Demange et al., 2021), we used methods that reveal subtle associations that may not be evident with *r*_g_. Collectively, this approach reveals the shared and distinct genetic pathways that link EA with substance use behaviors and enhances our understanding of the role of educational, cognitive, and noncognitive factors in shaping individuals’ health behaviors.

## Methods

### Summary statistics

We conducted secondary analyses of summary statistics from large GWAS of European-like ancestry (EUR) individuals for AC (*N* = 296,989) (Kember et al., 2023), AUD (*N* = 296,989, *N*_cases_ = 53,458) (Kember et al., 2023), CanUse (*N* = 162,082) (Pasman et al., 2018), and CUD (*N* = 445,847, *N*_cases_ = 22,260) (Levey et al., 2023; Pasman et al., 2018). The original studies received ethical approval and obtained informed consent from all participants. In the Million Veteran Program, AC was assessed using the Alcohol Use Disorders Identification Test-Consumption score (Bush et al., 1998), while AUD and CUD diagnoses were based on International Classification of Diseases 9th Revision and 10th Revision codes. The CanUse GWAS (Pasman et al., 2018) comprised samples from the UK Biobank and International Cannabis Consortium, with each cohort providing self-reported lifetime cannabis use information.

GWAS summary statistics for EA were obtained (Okbay et al., 2022), excluding individuals from the UK Biobank and 23andMe to avoid sample overlap (*N* = 324,162). We also obtained summary statistics from a GWAS-by-subtraction of EA and cognitive performance (CogEA *N* = 257,700 and NonCogEA *N* = 510,795) (Demange et al., 2021). Notably, the CogEA GWAS included UK Biobank participants, contributing to modest sample overlap with CanUse. The linkage disequilibrium score regression (LDSC) bivariate intercept term for CogEA-CanUse indicated limited but non-negligible overlap (0.05, standard error [SE] = 0.01).

### Polygenic overlap

Before performing MiXeR, we calculated genetic correlations among the substance-related phenotypes using LDSC (Bulik-Sullivan et al., 2015). We then used MiXeR to estimate the overall polygenic overlap between substance-related traits and EA (Frei et al., 2019). We first used univariate models to estimate the polygenicity and discoverability of each trait (Holland et al., 2020). We ensured that the Akaike information criterion (AIC) values of the univariate models were positive, which indicates that there is adequate power for bivariate MiXeR. Bivariate models estimate the genetic overlap between traits, regardless of the direction of effect of causal variants. Specifically, MiXeR models the joint distribution of GWAS *Z*-scores using a bivariate Gaussian mixture framework, estimating the proportions of SNPs with null, trait-specific, or shared nonzero effects. The Dice coefficient estimates the proportion of the total number of causal SNPs that are shared. MiXeR has been demonstrated to be robust to sample overlap (Frei et al., 2019).

### Local *r*_**g**_

We used LAVA (Werme et al., 2022) to estimate the local *r*_g_ across 2,495 loci – each consisting of ~1-Mb blocks of the genome – with the 1000 Genomes Phase 3 EUR panel as a linkage disequilibrium (LD) reference (Auton et al., 2015). We used univariate association signals to estimate local SNP heritability and bivariate association signals to identify regions with significant local *r*_g_. For the univariate test, we applied a Bonferroni correction to account for multiple testing (*p* < 0.05/2,495), whereas for the bivariate test, we applied an FDR *p*-value correction. When performing LAVA, we used the LDSC bivariate intercept (0.05, SE = 0.01) to adjust for sample overlap between CogEA and CanUse, which both included UK Biobank participants.

### Joint SNP-level associations

To detect SNPs jointly associated with each pairwise combination of substance- and education-related traits, we conducted cond/conjFDR analyses (Smeland et al., 2020). First, we obtained conditional FDR (condFDR) estimates by conditioning the effect for one trait (e.g. AC) on the other trait (EA). This analysis re-ranks the test statistic for a primary trait based on the strength of association with a secondary trait. For each pair of traits, we performed condFDR twice by switching the primary and secondary traits to determine a conjFDR value, defined as the maximum of two condFDR values. This provides a conservative estimate of each SNP’s association with both traits. Test statistics were corrected using a genomic inflation control procedure that randomly prunes SNPs across 500 iterations using an LD *r*^2^ threshold of 0.1 (Smeland et al., 2020). Given that condFDR is sensitive to sample overlap, results for CogEA-CanUse should be interpreted cautiously.

### SNP annotation

We annotated independent loci and identified functional SNP effects using Functional Mapping and Annotation v1.7.0 (Watanabe, Taskesen, van Bochoven, & Posthuma, 2017). SNPs within an LD block distance of 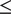 250 kb were merged. Independent significant SNPs were those with *r*^2^ < 0.6 and conjFDR < 0.05, whereas lead SNPs were those with *r*^2^ < 0.1. Candidate SNPs comprised all variants that had an LD *r*^2^ of 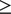 0.6 with an independent significant SNP. LD *r*^2^ values were obtained using the EUR 1000 Genomes Project Phase 3 reference panel. Gene-tissue expression analysis was performed with MAGMA v1.08 using GTEx v8 for adult tissue expression and BrainSpan for developmental tissue expression (de Leeuw, Mooij, Heskes, & Posthuma, 2015; The GTEx Consortium, 2020). Candidate SNPs were used as input for the gene-tissue expression analyses.

## Results

### Polygenic overlap

AC and AUD were strongly genetically correlated (*r*_g_ = 0.74, SE = 0.02, *p* = 2.31E-227). AC was also positively genetically correlated with CanUse (*r*_g_ = 0.29, SE = 0.04, *p* = 2.44E-12) and CUD (*r*_g_ = 0.48, SE = 0.04, *p* = 4.45E-28). Similarly, AUD was genetically correlated with CanUse (*r*_g_ = 0.28, SE = 0.04, *p* = 2.48E-12) and CUD (*r*_g_ = 0.82, SE = 0.03, 5.76E-124). CanUse and CUD were genetically correlated (*r*_g_ = 0.35, SE = 0.05, *p* = 2.56E-13), though to a lesser degree than AC and AUD.

**Figure 1.**
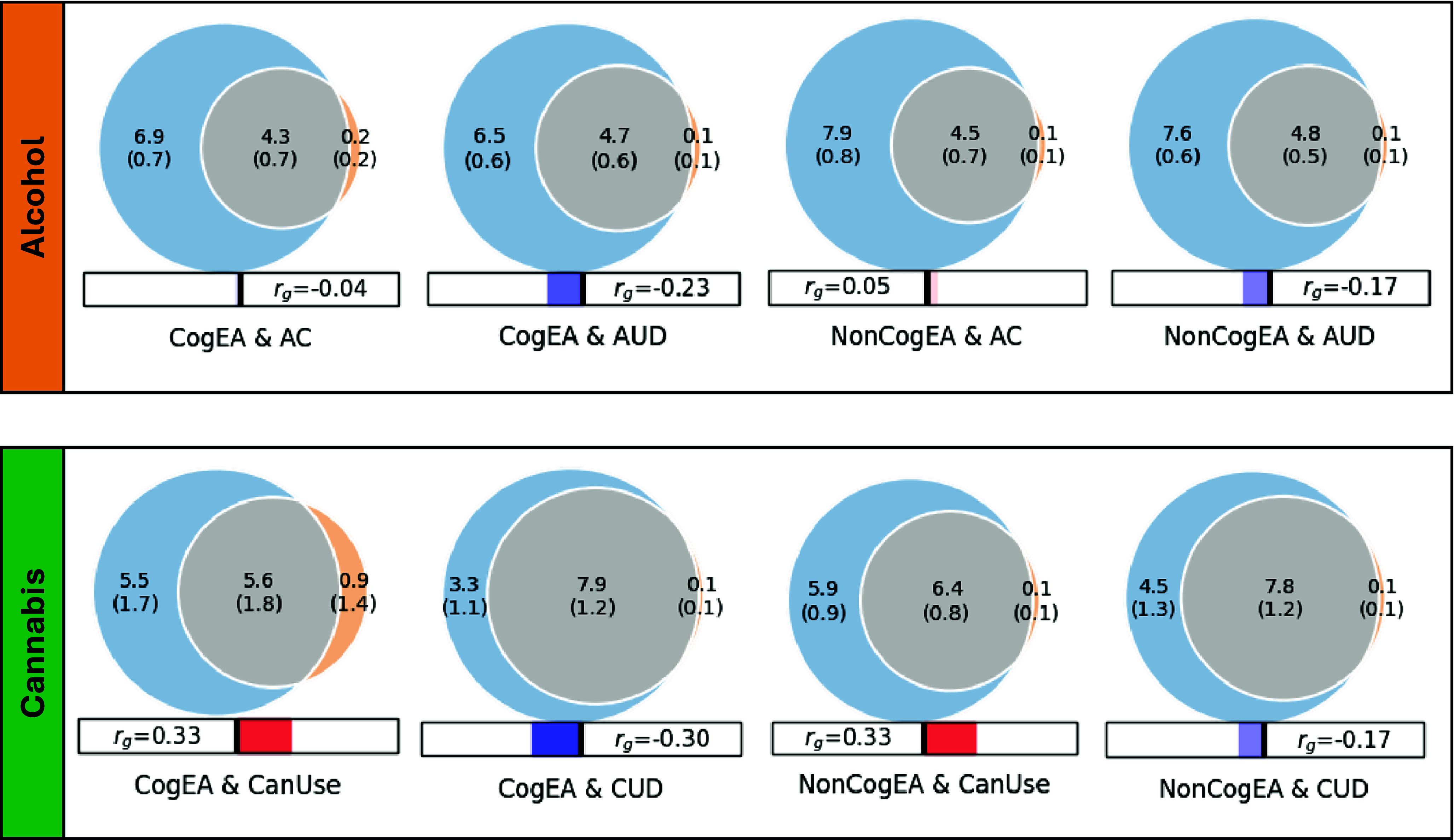
Results of bivariate causal mixture models (MiXeR). *Note:* CogEA = cognitive components of educational attainment, AC = alcohol consumption, AUD = alcohol use disorder, NonCogEA = noncognitive components of educational attainment, CanUse = lifetime cannabis use, CUD = cannabis use disorder, *r*_g_ = genetic correlation.

Univariate MiXeR models indicated that there was sufficient power to perform bivariate MiXeR analyses, as all AIC values were positive. Full univariate results are provided in Supplementary Table 1. Bivariate model results are shown in Figure 1. It is noteworthy that polygenic overlap and concordance estimates are reported as percentages for ease of interpretation, with standard deviations (SDs) reported as proportions.

**Alcohol.** EA shared a similar proportion of causal variants with AC (57.57%, SD = 0.07) and AUD (62.42%, SD = 0.04; Supplementary Figure 1). Despite similar polygenic overlap, over half of the shared variants for EA and AC had concordant effects (0.54, SD = 0.01), whereas concordance was lower for EA and AUD (0.37, SD = 0.01). CogEA and NonCogEA, which represent the cognitive and noncognitive components of EA, respectively, showed moderate polygenic overlap with AC and AUD. Like with EA, however, effect directions differed. CogEA and AC shared 54.85% (SD = 0.06) of their causal variants, of which 48.12% (SD = 0.01) were concordant in direction, while CogEA and AUD shared 58.65% (SD = 0.05), with only 38.40% (SD = 0.01) concordant. NonCogEA showed a similar degree of overlap with both AC and AUD (52.55% and 55.23%, respectively), but more shared variants were directionally consistent with AC (52.86%, SD = 0.01) than AUD (41.02%, SD = 0.01). Notably, the vast majority of causal genetic variants for AC and AUD were shared by EA, CogEA, or NonCogEA.

**Cannabis.** In contrast to the alcohol-related traits, there was greater divergence in the shared genetic effects of EA with CanUse and CUD. Although EA shared a lower proportion of causal variants with CanUse (48.07%, SD = 0.01) than CUD (84.18%, SD = 0.07; Supplementary Figure 2), the variants shared with CanUse were highly concordant (93.20%, SD = 0.03). Those shared with CUD, on the other hand, were much less concordant (38.30%, SD = 0.01). CogEA and NonCogEA exhibited a similar pattern. CogEA and CanUse shared 63.05% (SD = 0.18) of their causal variants, of which 71.42% (SD = 0.12) were directionally concordant. In contrast, for CogEA and CUD, although 81.66% of causal variants were shared, only 37.97% were concordant. Similarly, a smaller proportion of NonCogEA causal variants were shared with CanUse than with CUD (67.94%, SD = 0.06 vs. 76.67%, SD = 0.07), but a larger proportion of the variants shared with CanUse had concordant effect directions (65.56%, SD = 0.01 vs. 42.23%, SD = 0.01). Although CanUse exhibited more causal genetic variants that were not shared with EA (~2.5k of 6.5k variants) than the other substance-related traits, most genetic variants for CanUse and CUD were shared with EA, CogEA, and NonCogEA.

### Local *r*_**g**_

**Figure 2.**
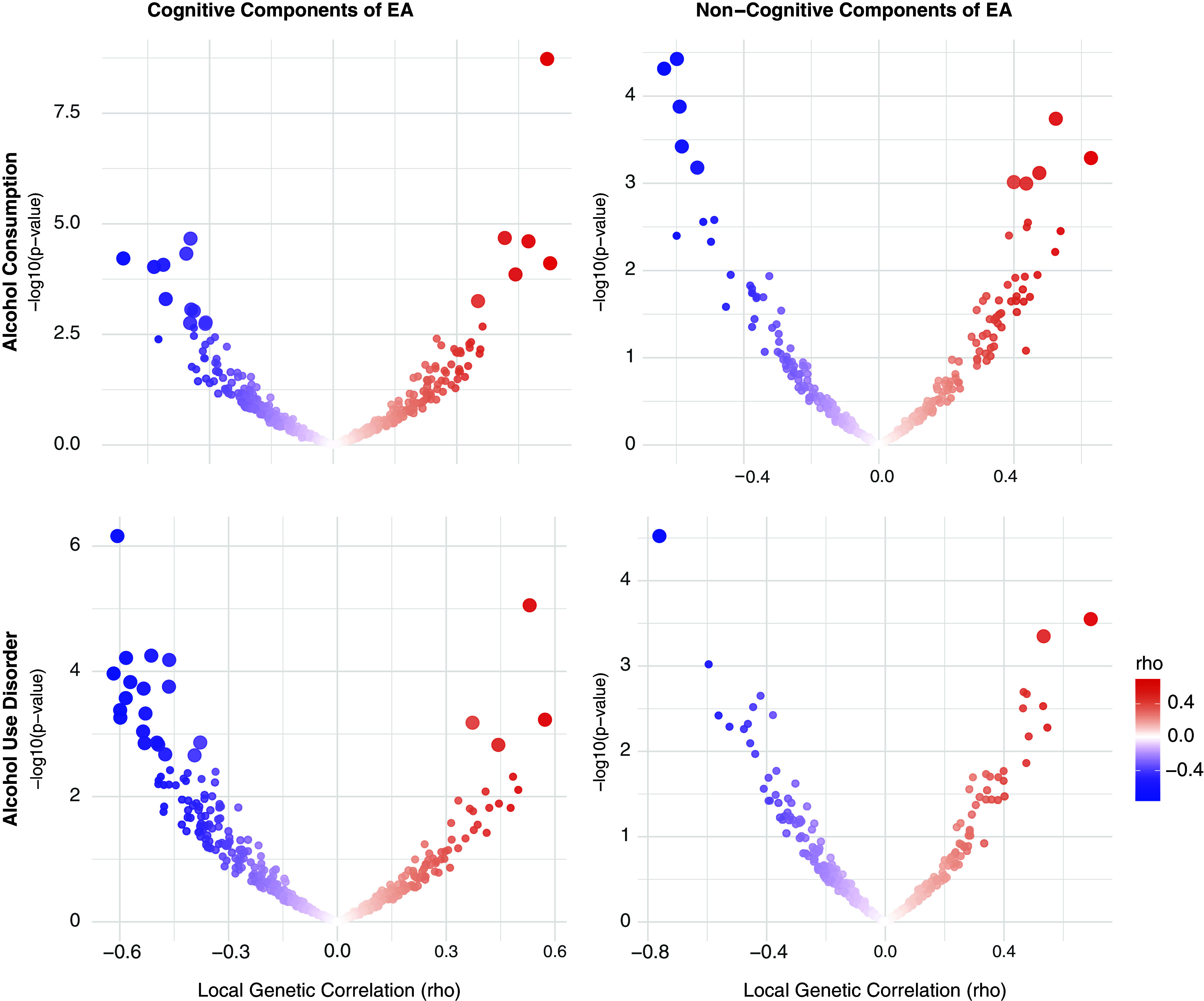
Volcano plots of local genetic correlations for alcohol consumption and use disorder. *Note:* EA = educational attainment. Larger dots represent significant local genetic correlations.

**Alcohol.** Using LAVA, EA had significant local *r*_g_ with AC at 7 loci and with AUD at 6 loci, with one region (chr 14:99474534-100786189) overlapping between the two alcohol-related traits. Across all loci tested, most (66.86%) showed a concordant direction of *r*_g_ between EA and both AC and AUD. When examining EA subcomponents, CogEA showed more widespread local *r*_g_, including at 17 loci with AC and 23 loci with AUD. Five loci overlapped across the two alcohol-related traits and had the same direction of *r*_g_. Overall, 67.03% of all loci tested had a concordant direction of *r*_g_ across AC and AUD. NonCogEA showed fewer significant local *r*_g_ (Figure 2): 10 for AC and 3 for AUD, with no overlap. Nevertheless, most loci (63.32%) were concordant in direction of *r*_g_ across both alcohol traits. Full results are provided in Supplementary Tables 2–7.

**Figure 3.**
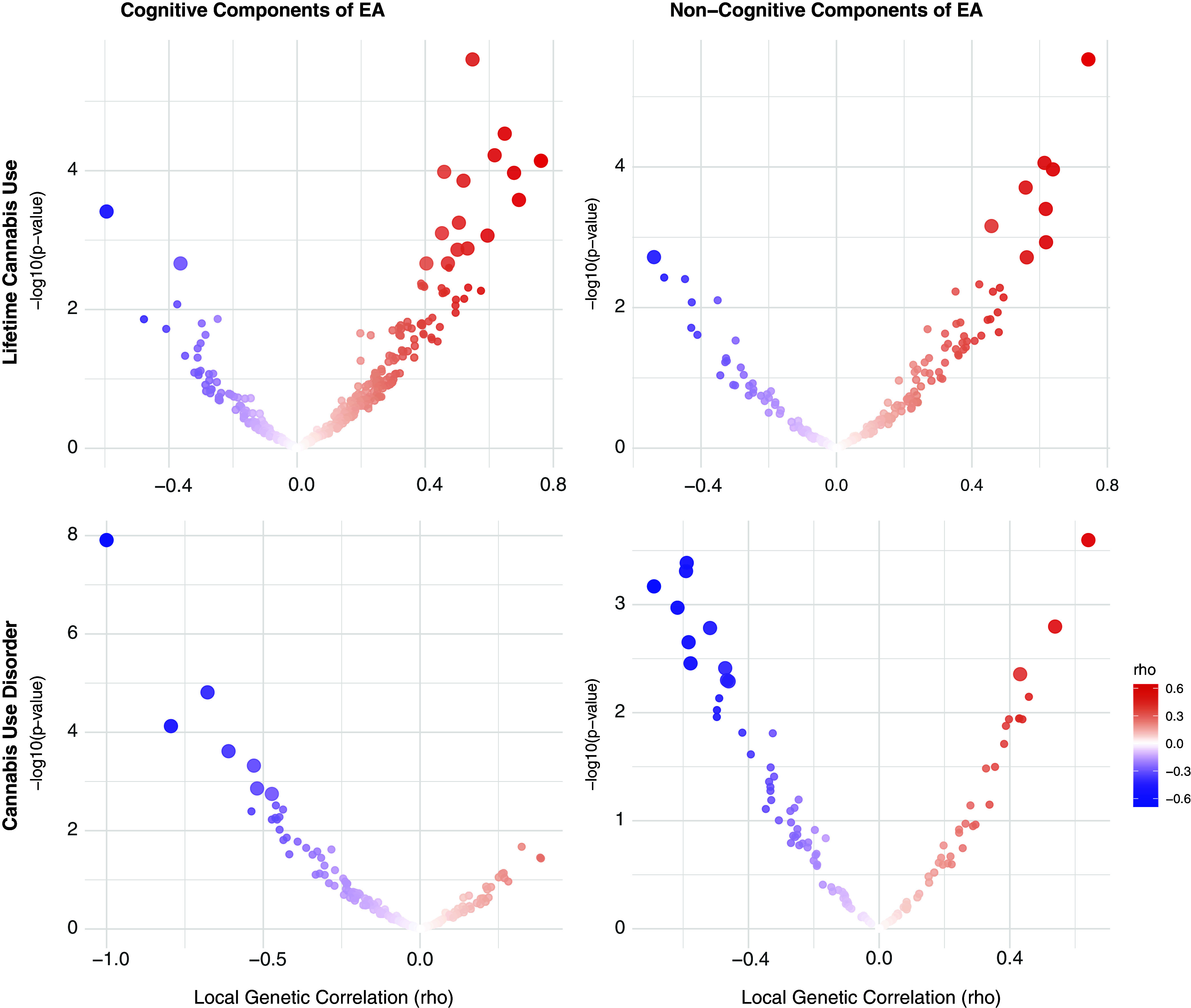
Volcano plots of local genetic correlations for lifetime cannabis use and disorder. *Note:* EA = educational attainment. Larger dots represent significant local genetic correlations.

**Cannabis.** For cannabis traits, EA had significant *r*_g_ with CanUse at 17 loci and with CUD at 5 loci, though none of these loci were shared across the two cannabis-related traits. Just under half (44.64%) of all loci tested showed a concordant direction of *r*_g_ between EA and both CanUse and CUD, reflecting more divergence in local effects than for alcohol-related traits. For CogEA, there was significant local *r*_g_ with CanUse at 17 loci and with CUD at 7 loci, again with no overlap between the two. There were concordant directions of *r*_g_ for both cannabis traits with CogEA at 39.53% of loci. NonCogEA showed local *r*_g_ with CanUse at 9 loci and with CUD at 13, with no overlap. Concordance across the two cannabis-related traits with NonCogEA (42.59%) was slightly higher than for CogEA (39.53%). See Figure 3 for a summary of the results. Full results are in Supplementary Tables 8–13.

### Joint SNP-level associations

**Figure 4.**
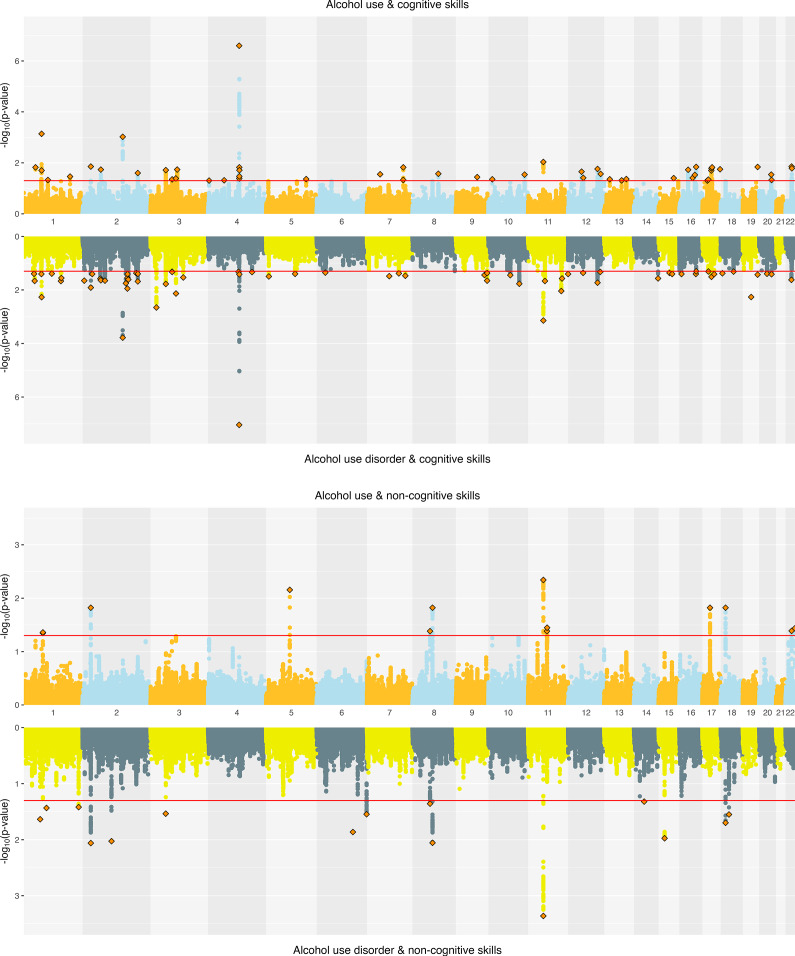
Miami plots of alcohol-related traits and educational attainment components.

**Alcohol.** ConjFDR analyses identified substantial overlap at the variant level between EA and alcohol-related traits. EA and AC had 108 jointly associated lead SNPs, of which 57 were concordant in effect direction. EA and AUD had 94 jointly associated SNPs, with 45 directionally concordant (Supplementary Figure 3). For CogEA, AC had 52 jointly associated lead SNPs (21 directionally concordant), while AUD had 67 (44 concordant). In contrast, NonCogEA had fewer joint associations with alcohol-related traits: 13 with AC (6 concordant) and 15 with AUD (6 concordant; Figure 4). Lead SNPs for the alcohol- and education-related trait pairs are in Supplementary Tables 14 and 15, and Supplementary Figures 5–7 show gene-based Manhattan plots.

**Figure 5.**
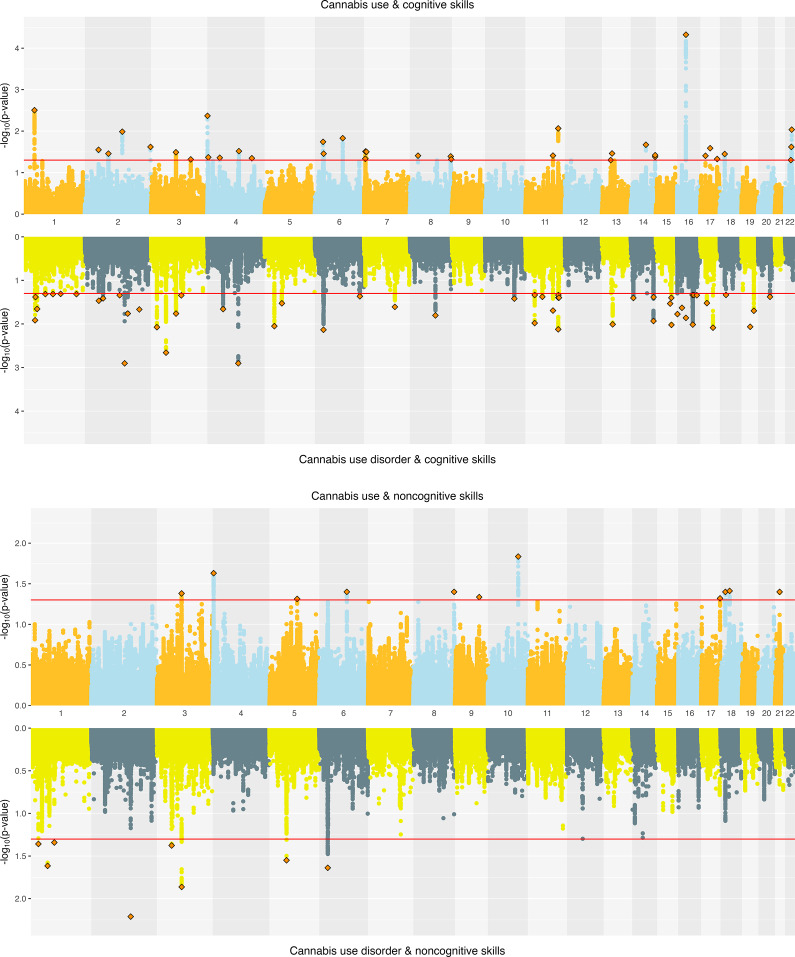
Miami plots of cannabis-related traits and educational attainment components.

**Cannabis.** Joint SNP-level associations were less abundant for cannabis- than alcohol-related traits but showed clearer separation between CanUse and CUD. EA and CanUse had 41 jointly associated SNPs, with 34 directionally concordant. EA and CUD had 28 jointly associated SNPs, with just 11 directionally concordant (Supplementary Figure 4). CogEA and CanUse had 36 jointly associated SNPs (13 concordant), while CogEA and CUD had 54 jointly associated SNPs (44 concordant), indicating greater overlap with CUD than CanUse. NonCogEA and CanUse had 11 jointly associated SNPs (4 concordant), while NonCogEA and CUD had 8 jointly associated SNPs (7 concordant; Figure 5), showing modest but highly directionally consistent associations of NonCogEA with CUD. Lead SNPs for the cannabis- and education-related trait pairs are in Supplementary Tables 16 and 17, and Supplementary Figures 8–10 show gene-based Manhattan plots.

### Functional annotation

**Alcohol.** SNPs jointly associated with EA and both AC and AUD were enriched for expression in the brain during early, early-mid, and late-mid prenatal development. There was no significant enrichment for SNPs jointly associated with the alcohol traits and CogEA or NonCogEA (Supplementary Figure 11). Analyses using MAGMA GTEx v8 tissues showed significant enrichment in brain and pituitary tissues for SNPs jointly associated with AUD and CogEA (Supplementary Figure 12), specifically in the nucleus accumbens (NAcc), hypothalamus, and caudate (Supplementary Figure 13).

**Cannabis.** For cannabis traits, only SNPs jointly associated with CanUse and EA showed significant enrichment during brain development in the early-mid and late-mid prenatal stages. No significant enrichment was identified for other jointly associated SNPs (Supplementary Figure 11). MAGMA tissue expression analysis showed significant SNP enrichment in skin tissues for CanUse and EA, in uterine cervix tissues for CanUse and CogEA, and in nerve tissues for CUD and CogEA (Supplementary Figure 12). The analysis across 54 tissues added specificity to the findings. For CanUse and EA, there was significant enrichment in the sun-exposed skin of the lower leg, and for CUD and CogEA, there was enrichment in the cervical spine and substantia nigra brain tissues. For CanUse and NonCogEA, there was enrichment in the cerebellum and its hemispheres (Supplementary Figure 13).

## Discussion

Across methods of increasing genetic specificity, our results provide insights into the paradox that EA is positively genetically correlated with the use of alcohol or cannabis but negatively correlated with AUD and CUD. These directionally opposite *r*_g_ cannot be explained by differences in overall polygenic overlap, as EA has greater polygenic overlap with AUD and CUD (62.42% and 84.18%, respectively) than with AC and CanUse (57.57% and 48.07%, respectively). However, the effect direction of the shared variants highlighted a key distinction. Variants shared between EA and SUDs more often had opposing effects on the two traits, while those shared with substance use had greater concordance.

Disaggregating EA into its cognitive (CogEA) and noncognitive (NonCogEA) components clarified this distinction across levels of specificity, with important nuance across methods. At the global level, MiXeR estimated higher genome-wide concordance for CogEA with AC and CanUse than with AUD and CUD, consistent with the findings for EA overall. In contrast, conjFDR showed that the effect direction of jointly associated SNPs was more often concordant for CogEA and AUD/CUD than for CogEA and AC/CanUse. The discrepancy across the two methods is likely due to differences in scope. Whereas MiXeR estimates the polygenic overlap and concordance across *all* causal variants (including many with small effects), conjFDR identifies the SNPs that are most strongly jointly associated. Thus, although many variants positively associated with CogEA protect against developing AUD and CUD, a smaller set of variants with strong effects *increases* the risk for these disorders.

For NonCogEA, there were also differences across methods. MiXeR analyses showed higher concordance of NonCogEA with AC and CanUse than with AUD and CUD. For cannabis traits, however, conjFDR analysis showed the opposite pattern. SNPs jointly associated with NonCogEA and CUD were more directionally concordant than those jointly associated with NonCogEA and CanUse. Thus, although genetic variants associated with noncognitive traits promote cannabis use generally, the most strongly associated variants may disproportionately contribute to risk for developing CUD. Variants may influence both NonCogEA (increasing liability for EA through noncognitive traits) and CUD via risk-taking or socially adaptive forms of disinhibition (Moncel et al., 2025). In competitive academic environments, these tendencies facilitate goal pursuit and strategic risk-taking. For example, research shows that individuals in leadership positions often have elevated dominance (Lilienfeld et al., 2012, 2014) and that risk-taking is associated with better academic outcomes (Petzel & Casad, 2022). However, in other contexts, these same traits can confer vulnerability to CUD.

At the local level, results from LAVA showed that genomic regions where EA has local *r*_g_ with AC/CanUse are largely distinct from those where EA is correlated with AUD/CUD. This pattern was consistent regardless of whether EA was considered as a whole or partitioned into cognitive and noncognitive components. Previous research has also shown that experimentation with substances and the development of SUD are shaped by at least partially distinct etiological factors (Lynskey et al., 2003; Richmond-Rakerd et al., 2016). The minimal overlap identified in LAVA analyses suggests that transitions from use to disorder may involve a shift in biological pathways, such as from those associated with greater opportunity or openness to use substances to those linked with compulsivity and executive dysfunction, key elements of SUDs. Thus, local differences in pleiotropy may, in addition to differences in effect direction, contribute to the paradoxical associations of EA and its subcomponents with alcohol and cannabis use, and AUD and CUD.

Analyses performed at the most granular level provided insight into the specific biological pathways that differentiate CanUse from CUD. SNP-based tissue enrichment showed that whereas SNPs jointly associated with CUD and CogEA were enriched in the substantia nigra, those jointly associated with CanUse and NonCogEA were enriched in the cerebellum and its hemispheres. Elevated dopamine function in the substantia nigra has been implicated in increased psychosis risk among individuals with CUD (Ahrens et al., 2025), and psychosis is often preceded by cognitive deficits (Jonas et al., 2022). These dopaminergic pathways in the substantia nigra may help explain the overlap between CUD and CogEA. In contrast, chronic cannabis use has been associated with altered cerebellar structure and poorer performance on noncognitive behavioral tasks that involve the cerebellum (Blithikioti et al., 2019). Thus, the potentially distinct neurobiological pathways underlying substance use and SUDs may also differ in their associations with cognitive and noncognitive traits. Although we also observed enrichment in several non-brain tissues, the relevance of these findings to cognition or substance-related behaviors is unclear and warrants cautious interpretation.

In contrast to the divergent effects between CanUse and CUD that were revealed by enrichment analyses, we found evidence of shared effects between AUD and AC. SNPs jointly associated with AUD and EA, as well as those associated with AC and EA, were linked to enriched expression in brain tissues during prenatal development. Prenatal stress plays a causal role in the development of SUDs later in life (Pastor, Antonelli, & Pallares, 2017) and is related to lower school achievement during childhood (Li et al., 2013). Our findings point to a shared genetic etiology that may account for this co-occurrence.

When EA was broken down into its subcomponents, SNPs jointly associated with AUD and CogEA showed enriched expression in the hypothalamus and two components of the basal ganglia – the NAcc and caudate nucleus. The basal ganglia have already been a target for intervention in AUD, with deep brain stimulation of the NAcc producing significant reductions in alcohol-related craving and compulsive behavior (Davidson et al., 2022). Other evidence supports the importance of these regions for understanding alcohol-related risk and cognitive functioning. For example, lower white matter integrity between the NAcc and prefrontal cortex is prospectively associated with earlier initiation of binge drinking (Morales et al., 2020). Similarly, the caudate nucleus is sensitive to prenatal alcohol exposure, with lower volume in this region predicting poorer cognitive control in individuals exposed to alcohol *in utero*, even after controlling for total brain volume, intelligence, and age (Fryer et al., 2012). Taken together, these findings suggest that genetic variants jointly associated with CogEA and AUD may influence risk by shaping the striatal circuits integral to balancing reward motivation with executive control.

Our study has several limitations. First, analyses were conducted only among EUR individuals due to the limited availability of GWAS in other population groups, which potentially limits the generalizability of our findings to other genetically inferred ancestry groups. Additionally, educational systems, access to education, and the relationship between education and health differ across societies (Eikemo, Huisman, Bambra, & Kunst, 2008). Thus, the genetic architecture linking EA and substance-related traits may vary across groups as well. Partitioning EA into CogEA and NonCogEA offered a more nuanced view of this complexity, but studies are needed that examine whether the observed patterns are consistent in other settings and groups. Second, the AUD and AC GWAS comprised predominantly of the Million Veteran Program participants, of whom the vast majority are male (~90%). Although sex-stratified GWAS of substance use traits are limited, recent efforts have identified several sex-specific genetic loci for alcohol use behaviors (Vilar-Ribó et al., 2025), and these GWAS could prove useful for future studies investigating shared genetic architecture with educational traits. A third consideration is that AC and CanUse reflect different stages of substance involvement and differ in their associations with AUD and CUD, respectively. This likely contributed to the clearer separation between CanUse and CUD in our results relative to AC and AUD, which often showed more similar associations with educational traits. A recently published GWAS of cannabis use frequency in 23andMe participants provides an opportunity for future exploration of these associations (Thorpe et al., 2025). Fourth, differences in GWAS sample sizes (e.g. for CogEA [*N* ≈ 258k] and NonCogEA [*N* ≈ 511k]) influence the power to detect polygenic overlap across the methods we used, with greater effects on SNP-level discovery than genome-wide estimates. Finally, although our analytic approach captured the overlap and divergence in genetic architecture for EA and substance-related traits, it does not provide insights into the causal directions of these associations.

By integrating multiple analyses of increasing genetic specificity, we show that the paradoxical genetic correlations between EA and alcohol/cannabis use versus AUD/CUD reflect more than just differences in overall polygenic overlap. Instead, a combination of differences in effect direction, associated genomic regions, and neurobiological pathways better explains the paradoxical associations. Partitioning EA into its cognitive and noncognitive components highlights how traits generally viewed as promoting educational success – including curiosity, openness, and cognitive ability – may also increase vulnerability to the development of SUDs, particularly CUD. These findings reinforce our understanding of EA as a multidimensional trait with complex links to substance-related behaviors.

## Supporting information

10.1017/S0033291726103353.sm001 Davis et al. supplementary material 1 Davis et al. supplementary material

10.1017/S0033291726103353.sm002 Davis et al. supplementary material 2 Davis et al. supplementary material

## Supplementary material

The supplementary material for this article can be found at http://doi.org/10.1017/S0033291726103353.
