# Supplementary material for: Education shares distinct genetic influences with substance use and disorder
Source: Psychol Med. 2026 Feb 2;56:e38. doi: 10.1017/S0033291726103353 (PMC12887118; doi:10.1017/S0033291726103353)
Supplement: Davis et al. supplementary material 1 — Davis et al. supplementary material [file S0033291726103353sup001.docx]

**Supplementary Materials**

Davis et al., Education shares distinct genetic influences with substance use and disorder

[Supplementary Figures 2](#_Toc217914856)

[**Supplementary Figure 1. Bivariate MiXeR results for educational attainment (EA) and alcohol-related traits.** 2](#_Toc217914857)

[**Supplementary Figure 2. Bivariate MiXeR results for educational attainment (EA) and cannabis-related traits.** 3](#_Toc217914858)

[**Supplementary Figure 3. Miami plots of alcohol-related traits and educational attainment.** 4](#_Toc217914859)

[**Supplementary Figure 4. Miami plots of cannabis-related traits and educational attainment** 5](#_Toc217914860)

[**Supplementary Figure 5. Gene-based Manhattan plot for alcohol-related traits with educational attainment.** 6](#_Toc217914861)

[**Supplementary Figure 6. Gene-based Manhattan plot for alcohol-related traits with the cognitive components of educational attainment.** 7](#_Toc217914862)

[**Supplementary Figure 7. Gene-based Manhattan plot for alcohol-related traits with the non-cognitive components of educational attainment.** 8](#_Toc217914863)

[**Supplementary Figure 8. Gene-based Manhattan plot for cannabis-related traits with educational attainment.** 9](#_Toc217914864)

[**Supplementary Figure 9. Gene-based Manhattan plot for cannabis-related traits with the cognitive aspects of educational attainment.** 10](#_Toc217914865)

[**Supplementary Figure 10. Gene-based Manhattan plot for cannabis-related traits with the non-cognitive aspects of educational attainment.** 11](#_Toc217914866)

[**Supplementary Figure 11. MAGMA results of BrainSpan 11 different developmental ages for each joint analysis.** 12](#_Toc217914867)

[**Supplementary Figure 12. MAGMA results of GTEx v8 30 tissue types for each joint analysis.** 13](#_Toc217914868)

[**Supplementary Figure 13. MAGMA results of GTEx v8 53 tissue types for each joint analysis.** 14](#_Toc217914869)

# **Supplementary Figures**


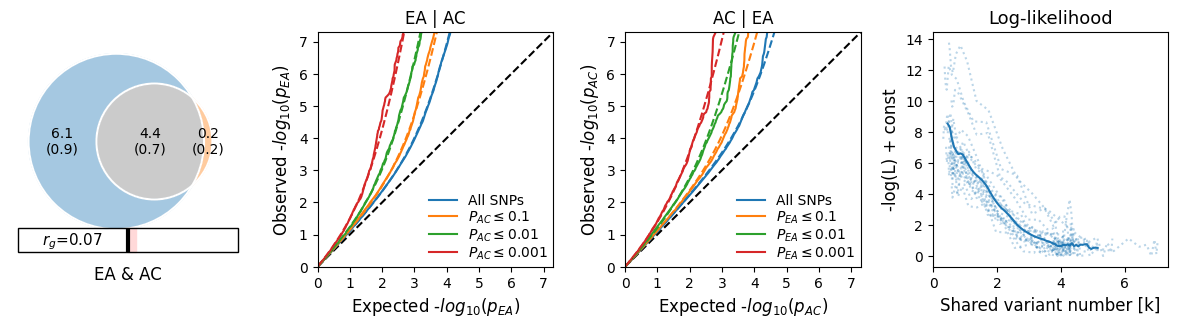


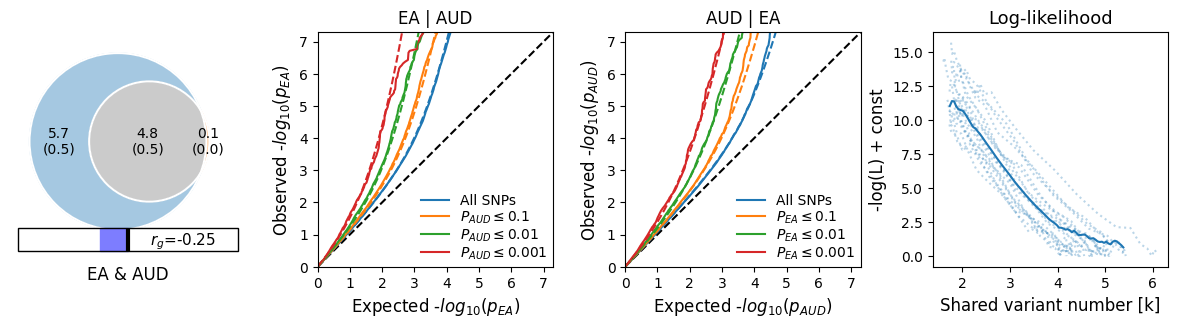


## **Supplementary Figure 1. Bivariate MiXeR results for educational attainment (EA) and alcohol-related traits.**

*Note:* EA = educational attainment, AC = alcohol consumption, AUD = alcohol use disorder.


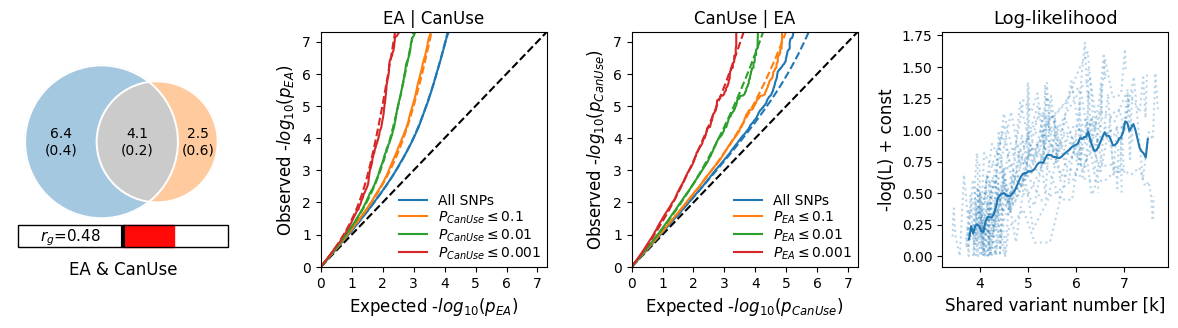


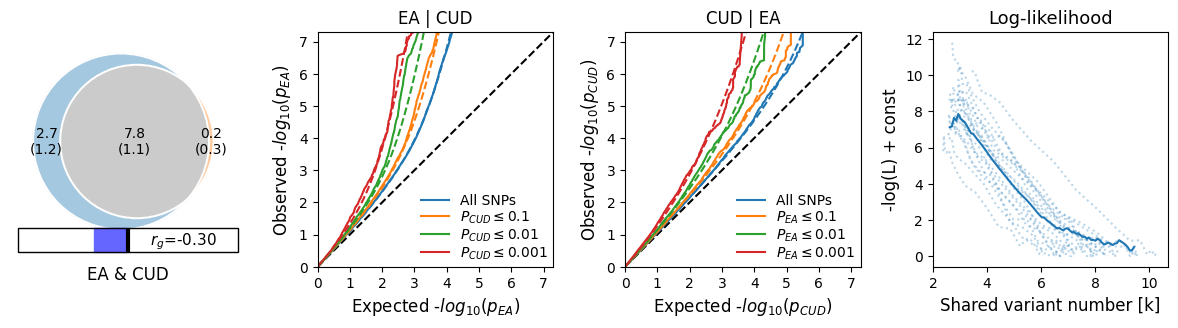


## **Supplementary Figure 2. Bivariate MiXeR results for educational attainment (EA) and cannabis-related traits.**

*Note:* EA = educational attainment, CanUse = lifetime cannabis use, CUD = cannabis use disorder.


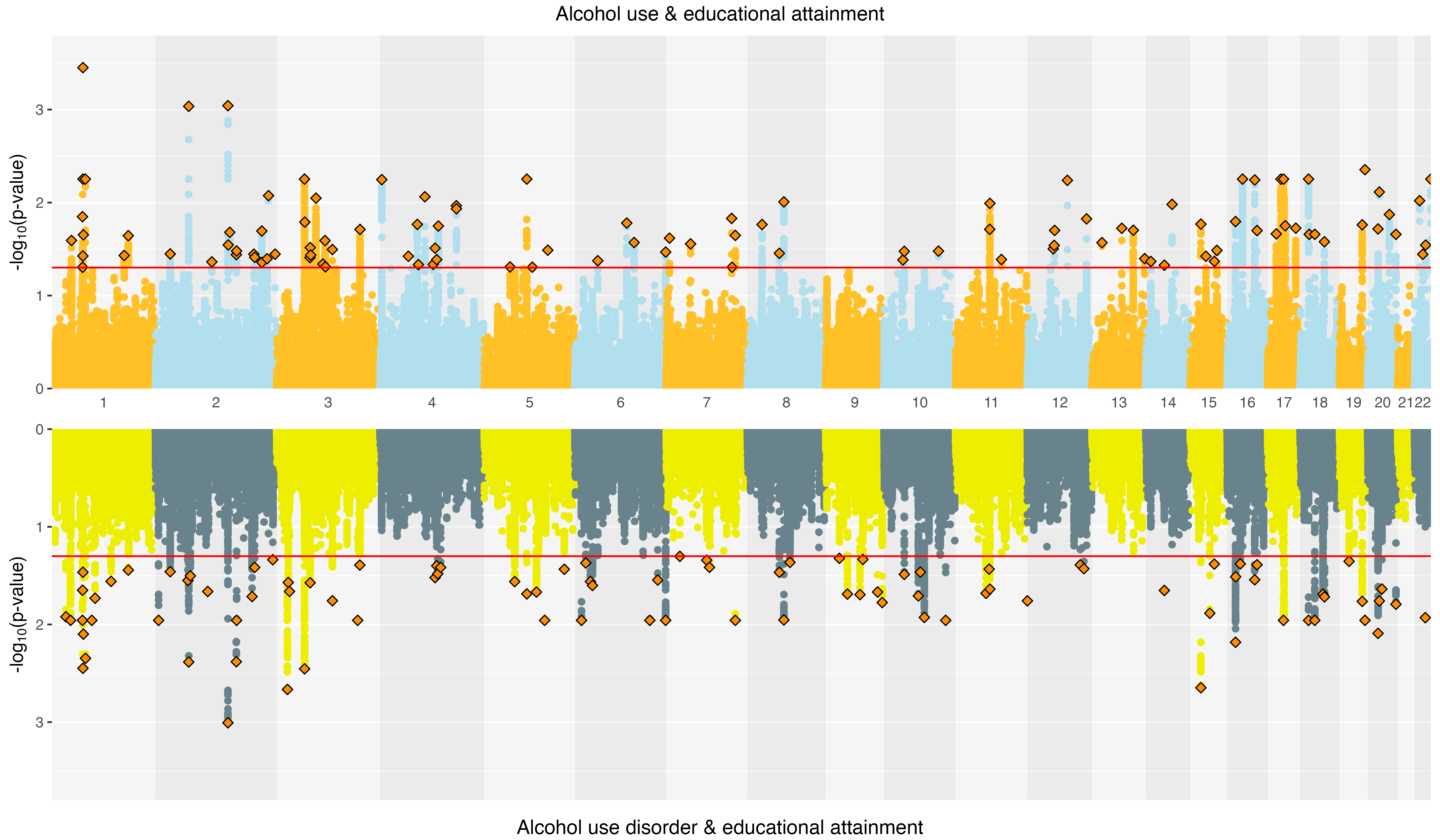


## **Supplementary Figure 3. Miami plots of alcohol-related traits and educational attainment.**

*Note*: Log_10_-transformed conjFDR values for each SNP are on the y axes and chromosomal positions are on the x axes. The horizontal line is the threshold for significant shared associations of alcohol use and alcohol use disorder with educational attainment (conjFDR <0.05). Independent lead SNPs are outlined diamonds.


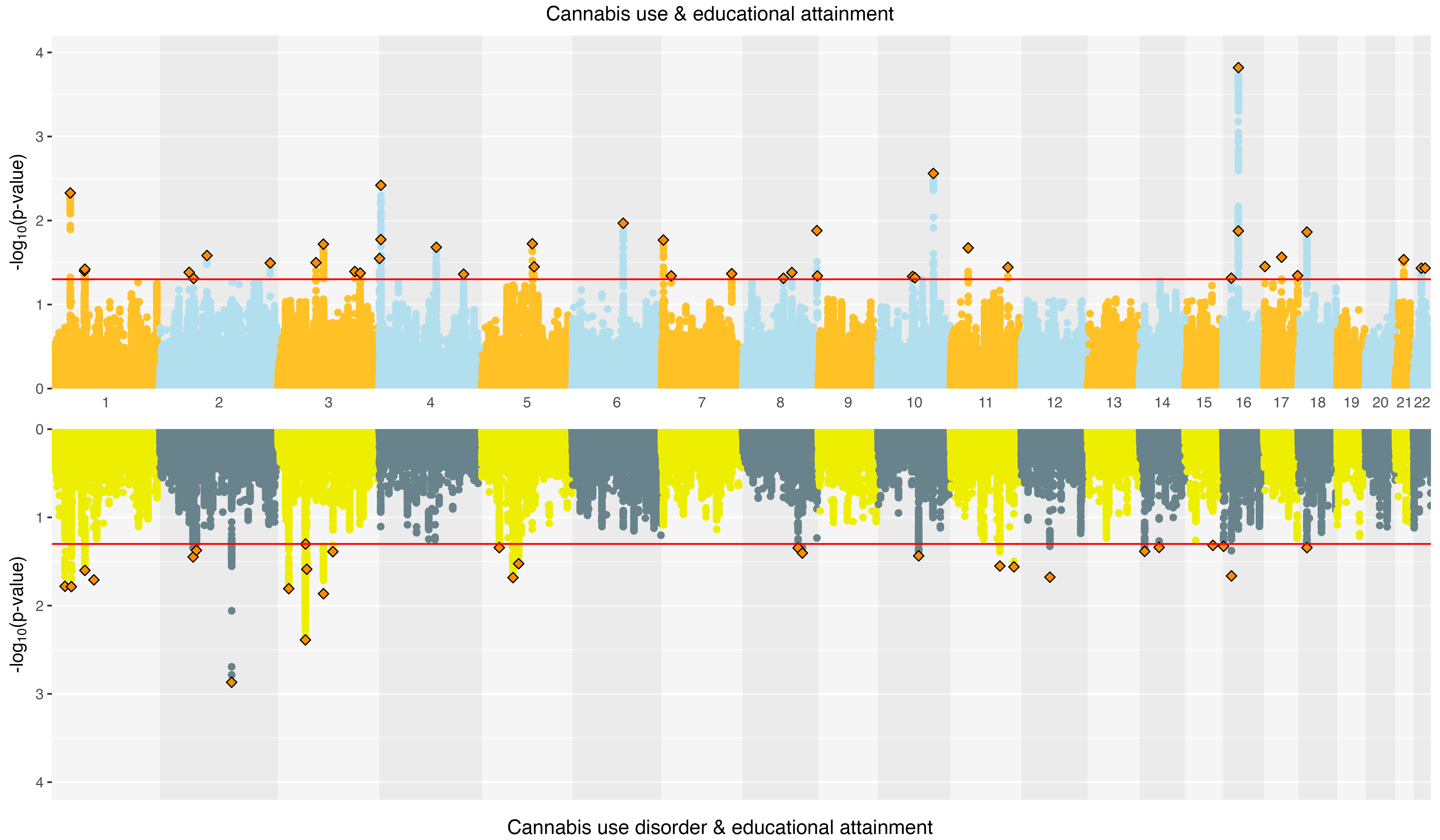


## **Supplementary Figure 4. Miami plots of cannabis-related traits and educational attainment**

*Note*: Log_10_-transformed conjFDR values for each SNP are on the y axes and chromosomal positions are on the x axes. The horizontal line is the threshold for significant shared associations of cannabis use and cannabis use disorder with educational attainment (conjFDR <0.05). Independent lead SNPs are outlined diamonds.


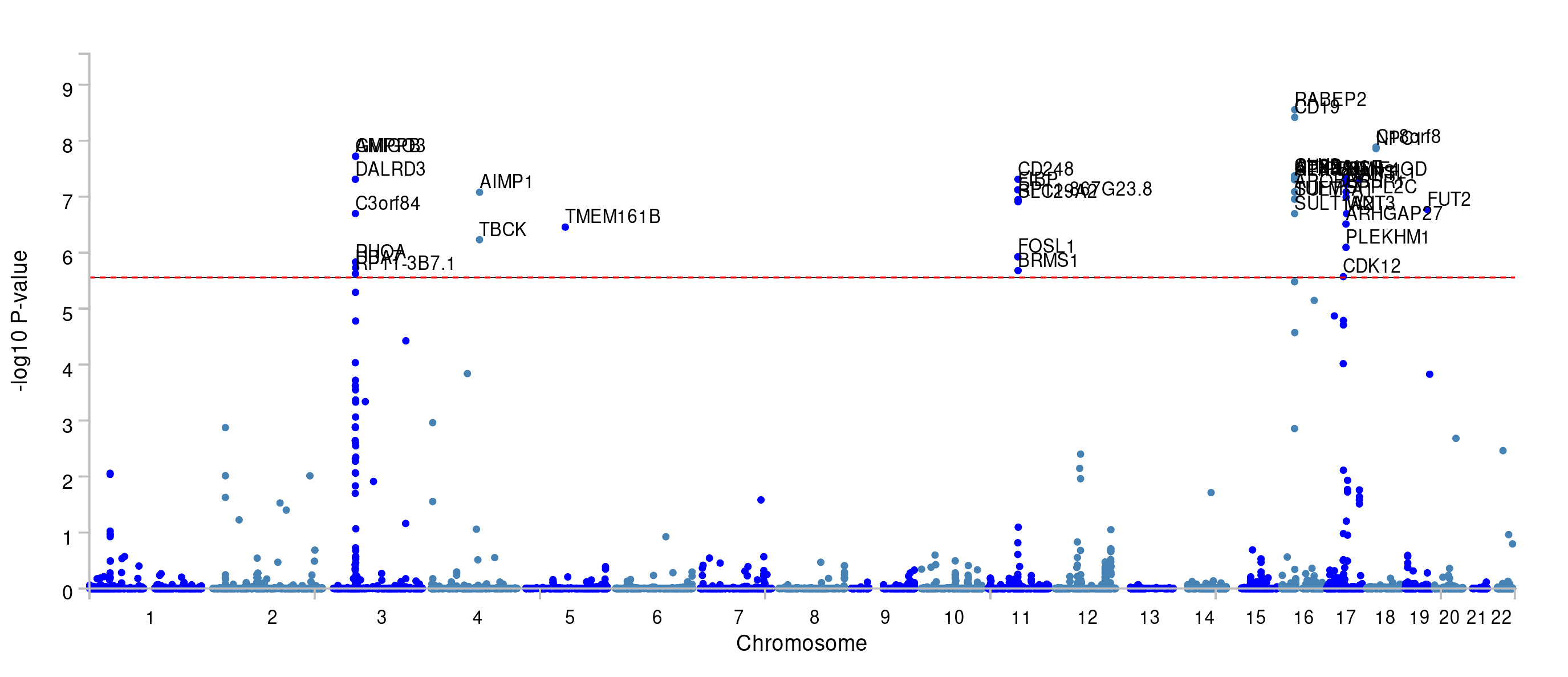


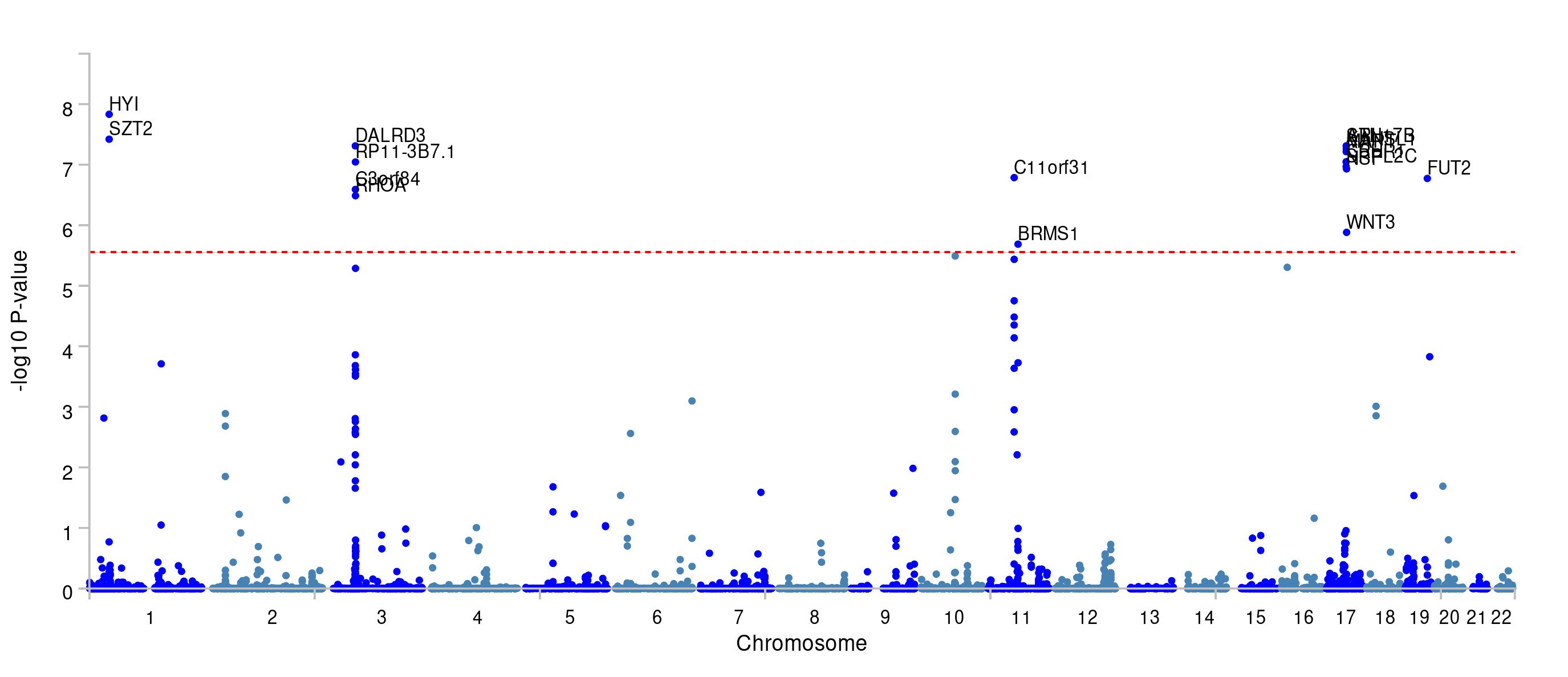


## **Supplementary Figure 5. Gene-based Manhattan plot for alcohol-related traits with educational attainment.**

*Note:* The top panel displays results for the joint SNP-level associations of alcohol consumption (AC) and educational attainment (EA), while the bottom panel shows results for alcohol use disorder (AUD) and EA.


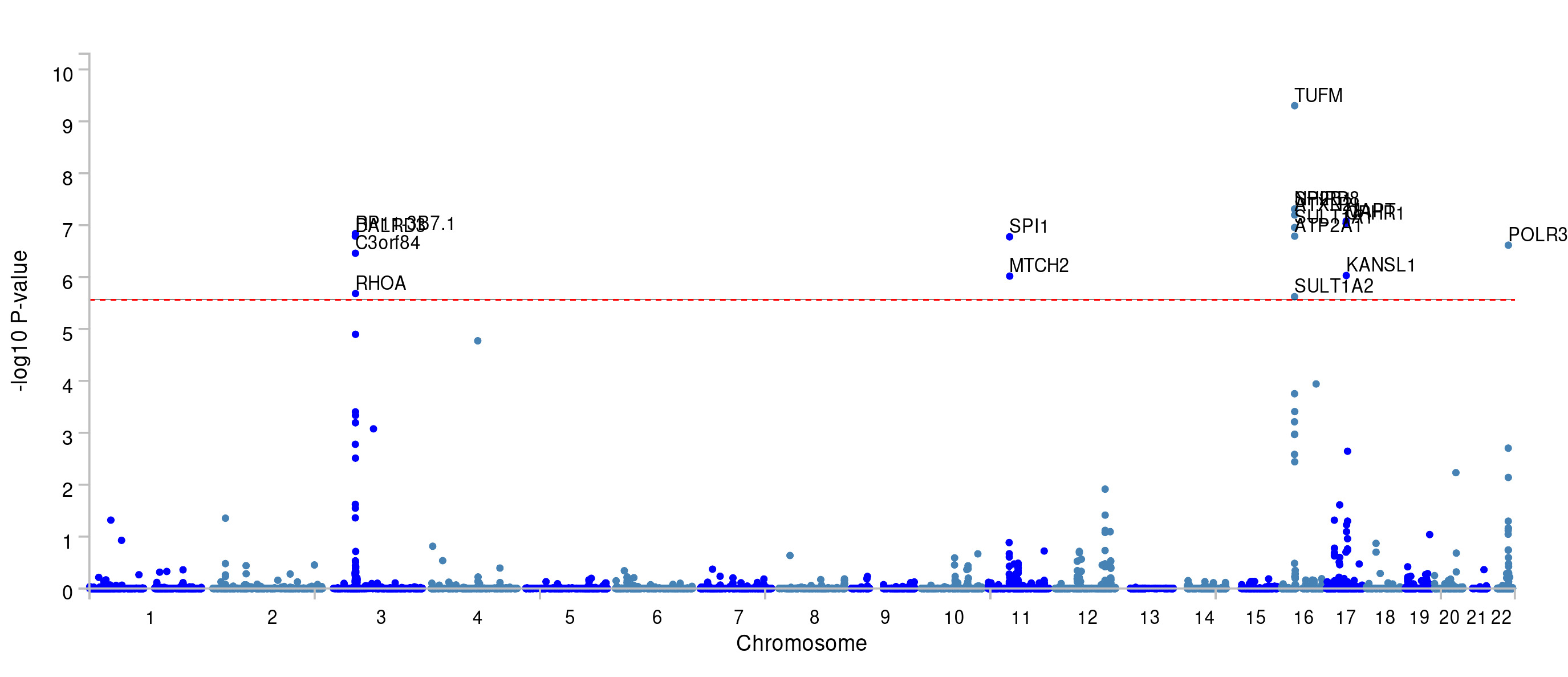


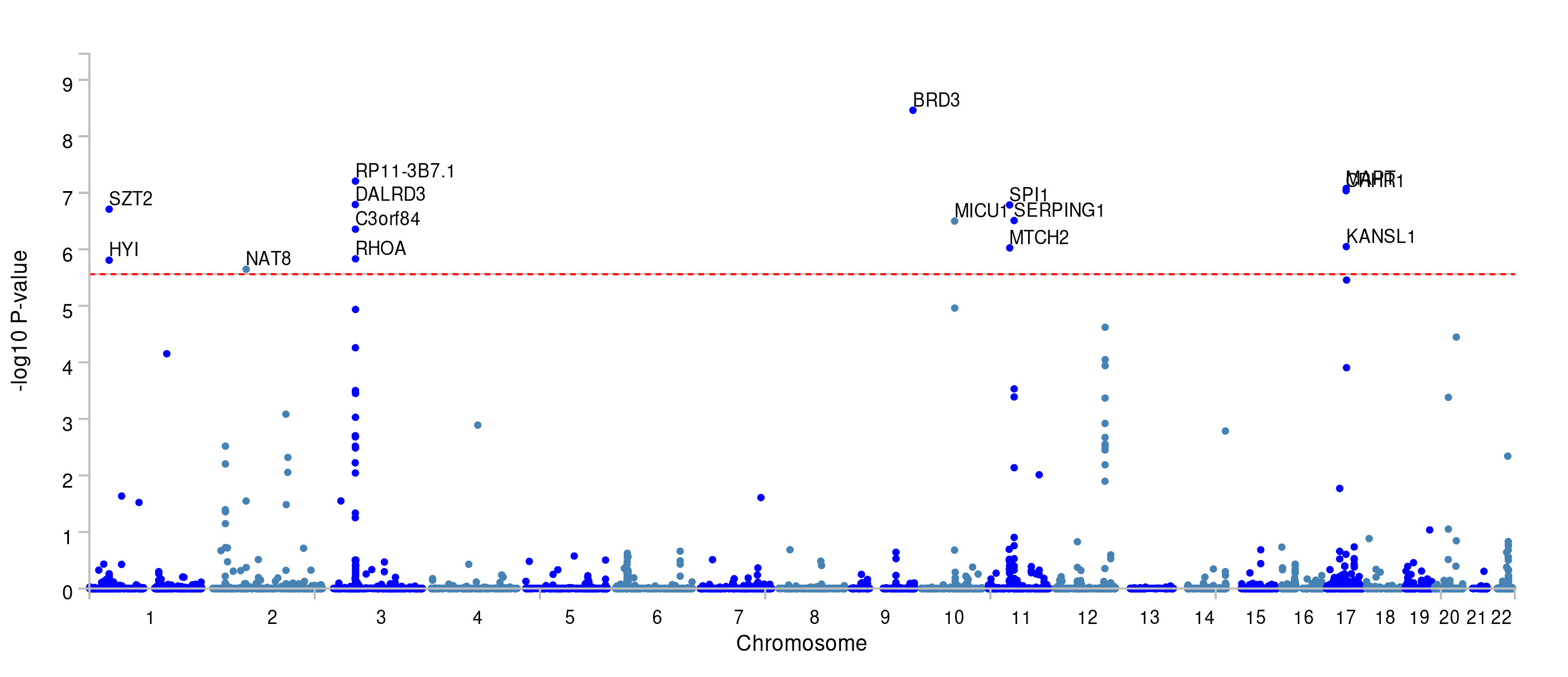


## **Supplementary Figure 6. Gene-based Manhattan plot for alcohol-related traits with the cognitive components of educational attainment.**

*Note:* The top panel displays results for the joint SNP-level associations of alcohol consumption (AC) and cognitive components of educational attainment (CogEA), while the bottom panel shows results for alcohol use disorder (AUD) and CogEA.


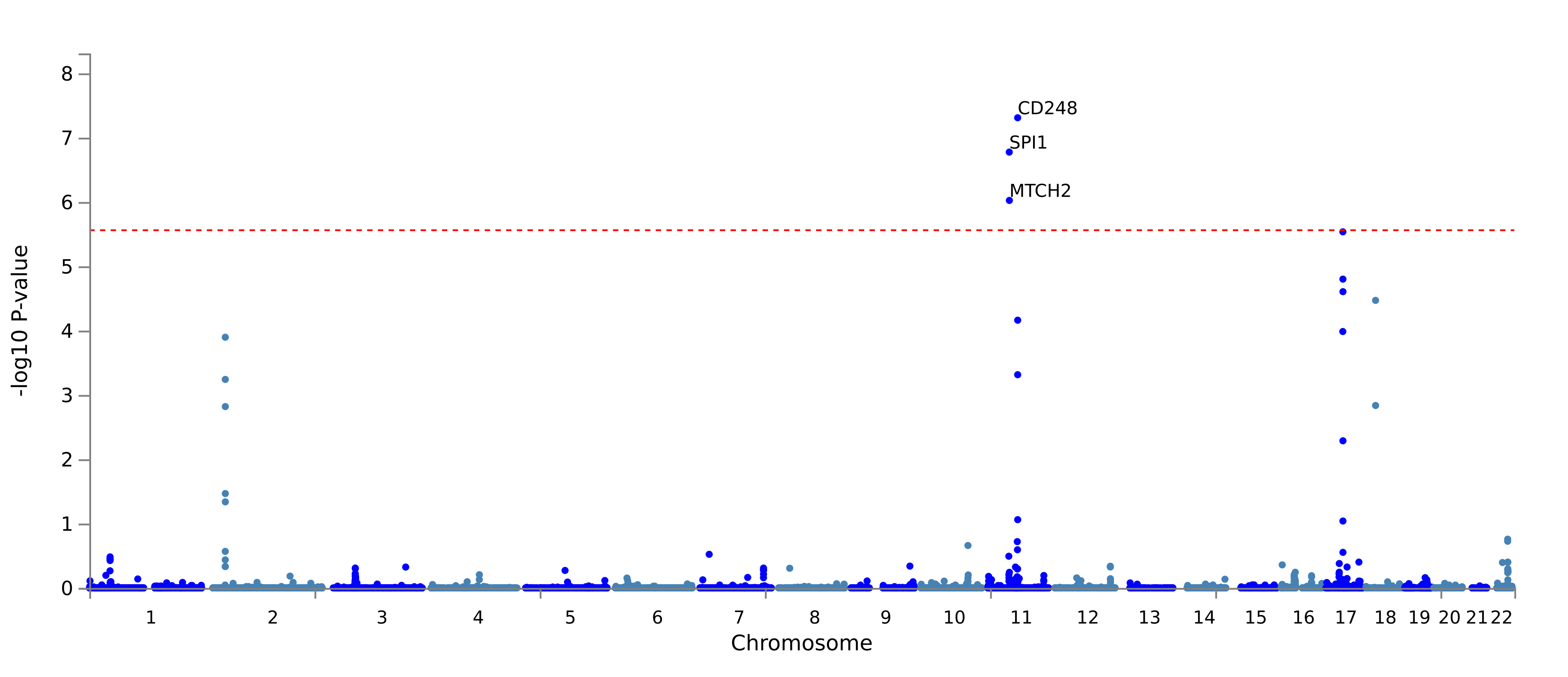


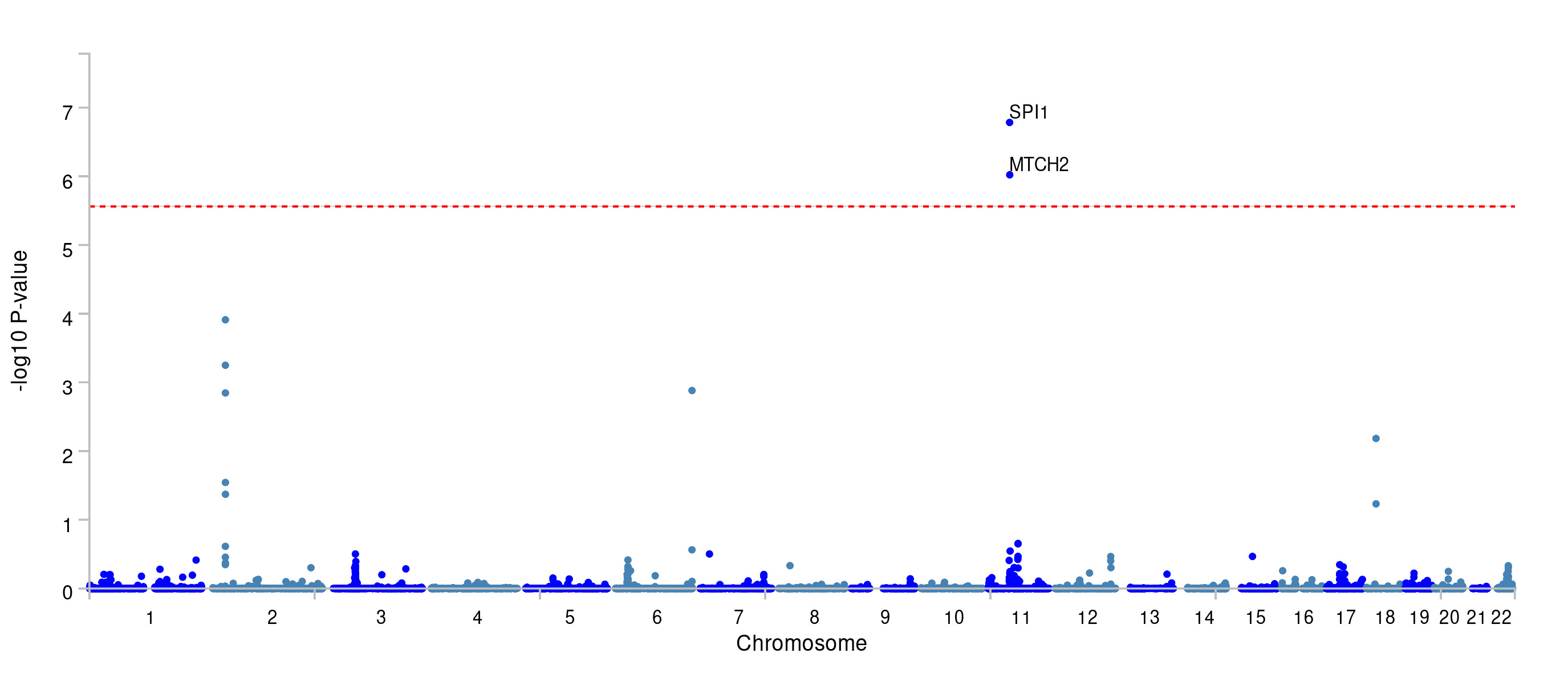


## **Supplementary Figure 7. Gene-based Manhattan plot for alcohol-related traits with the non-cognitive components of educational attainment.**

*Note:* The top panel displays results for the joint SNP-level associations of alcohol consumption (AC) and the non-cognitive components of educational attainment (NonCogEA), while the bottom panel shows results for alcohol use disorder (AUD) and NonCogEA.


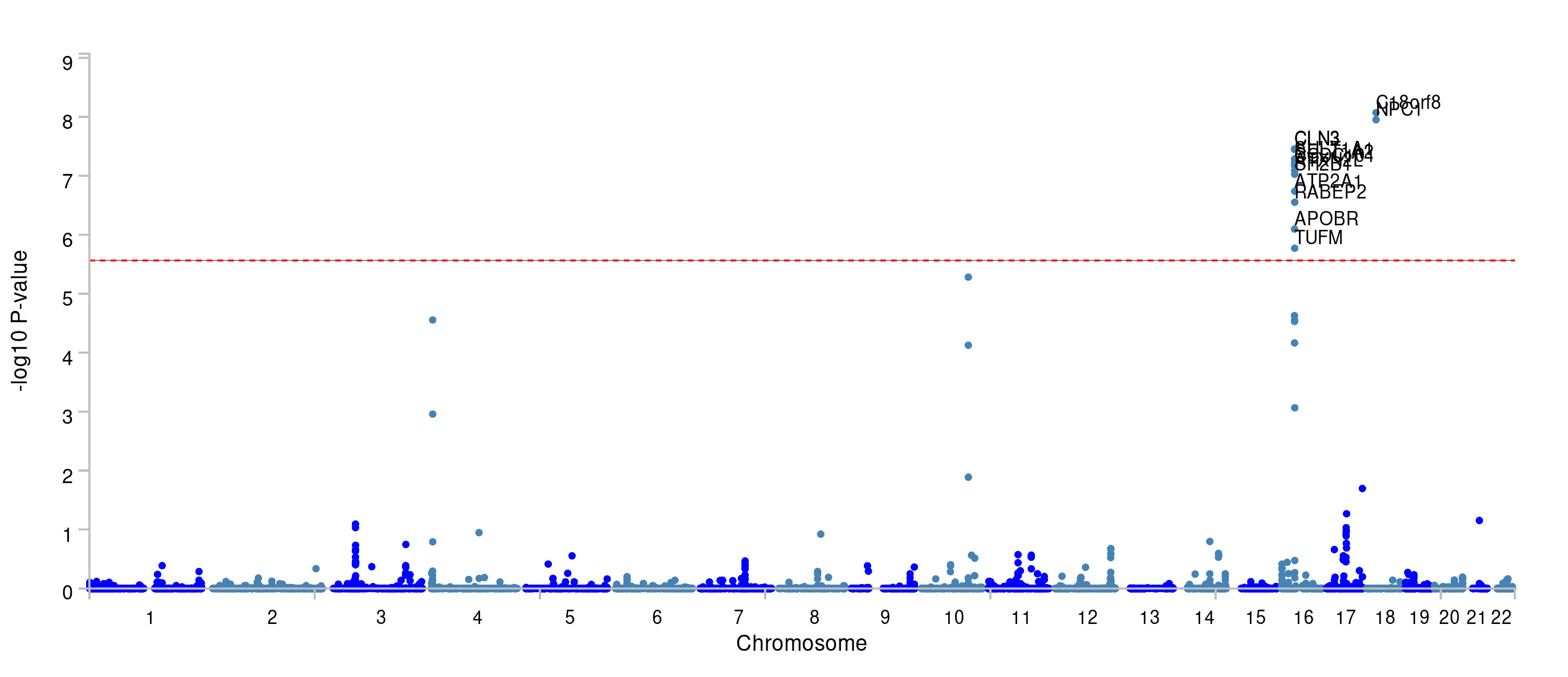


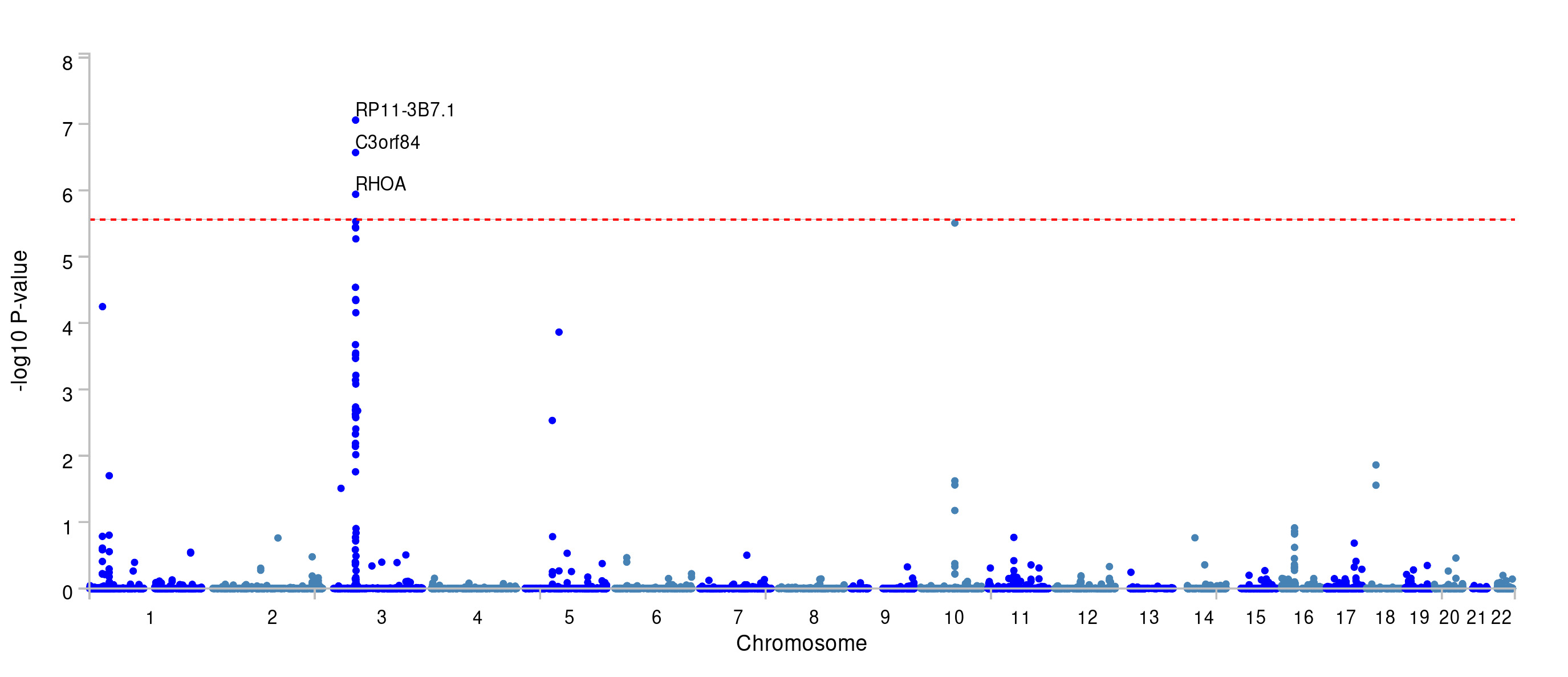


## **Supplementary Figure 8. Gene-based Manhattan plot for cannabis-related traits with educational attainment.**

*Note:* The top panel displays results for the joint SNP-level associations of lifetime cannabis use (CanUse) and educational attainment (EA), while the bottom panel shows results for cannabis use disorder (CUD) and EA.


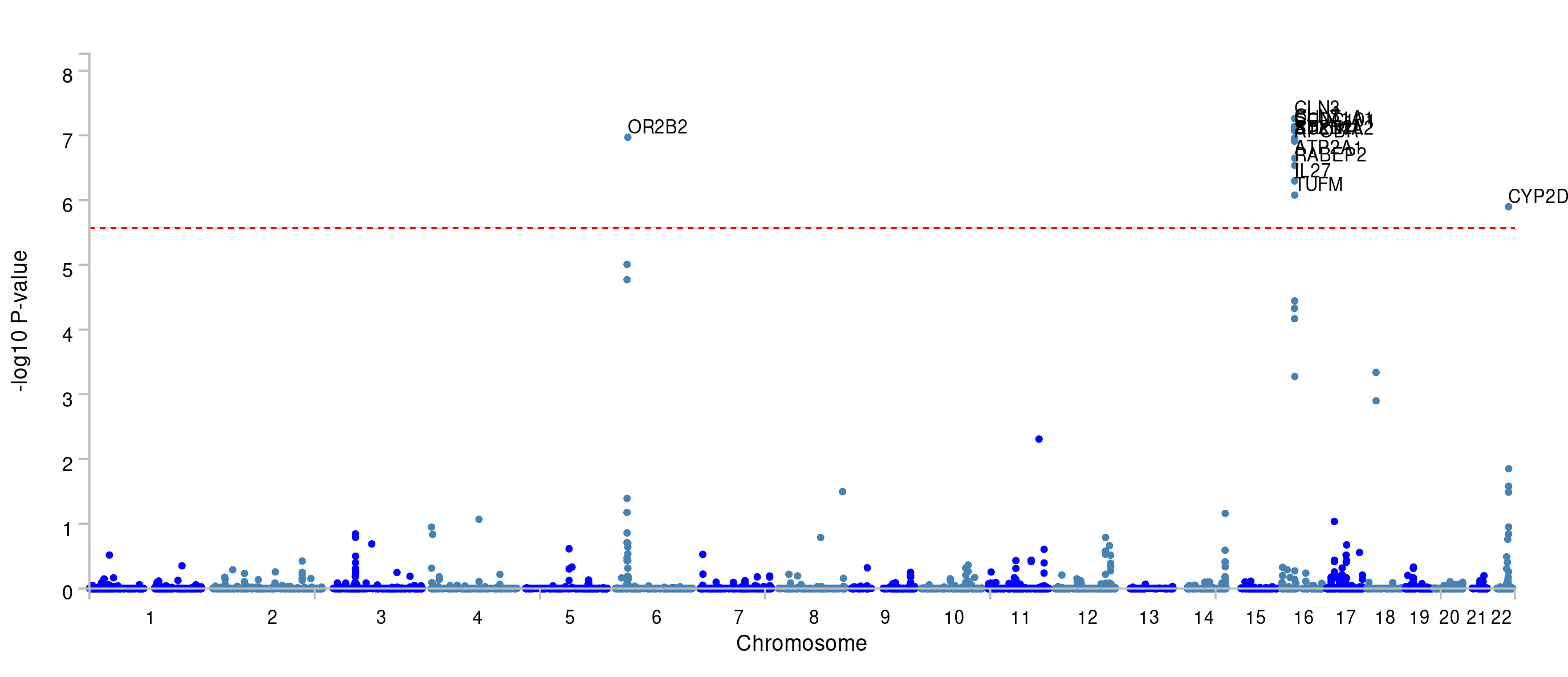


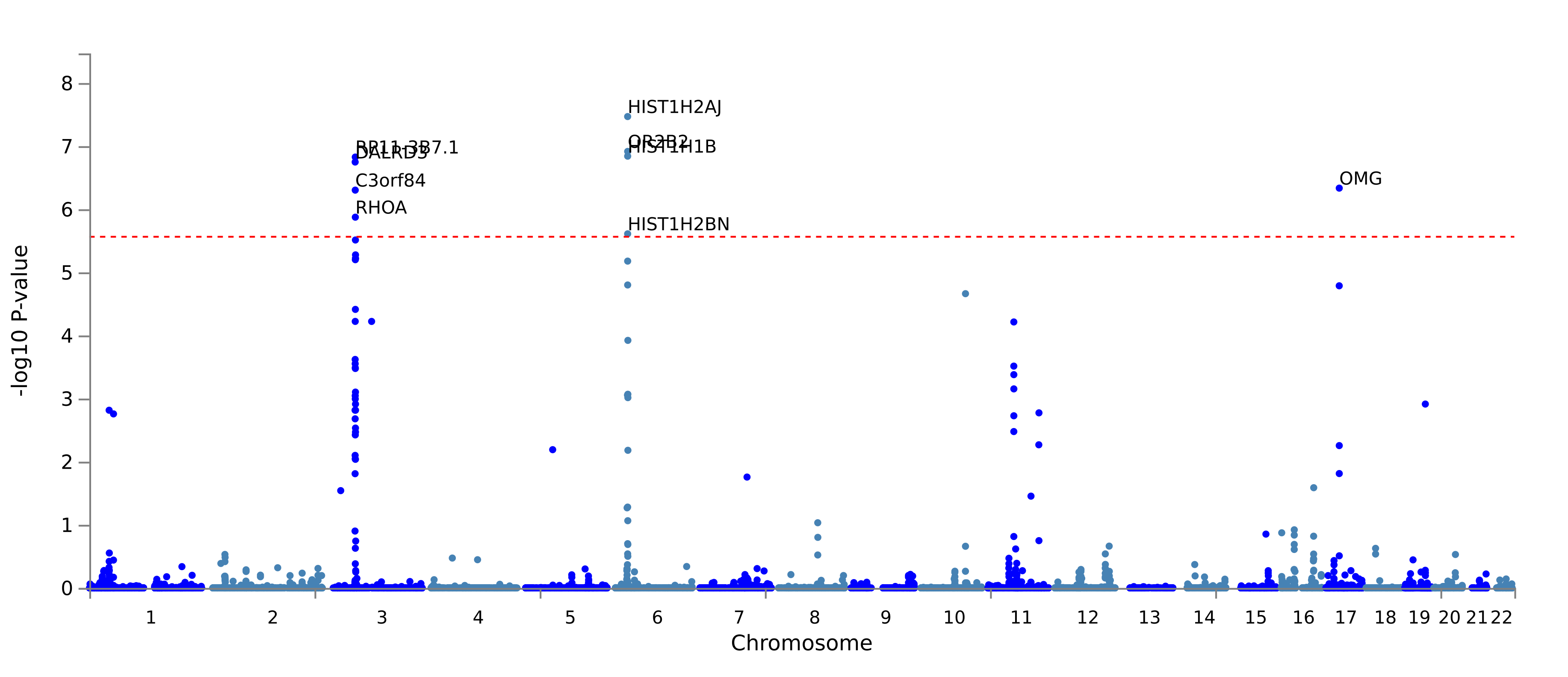


## **Supplementary Figure 9. Gene-based Manhattan plot for cannabis-related traits with the cognitive aspects of educational attainment.**

*Note:* The top panel displays results for the joint SNP-level associations of lifetime cannabis use (CanUse) and the cognitive components of educational attainment (CogEA), while the bottom panel shows results for cannabis use disorder (CUD) and CogEA.


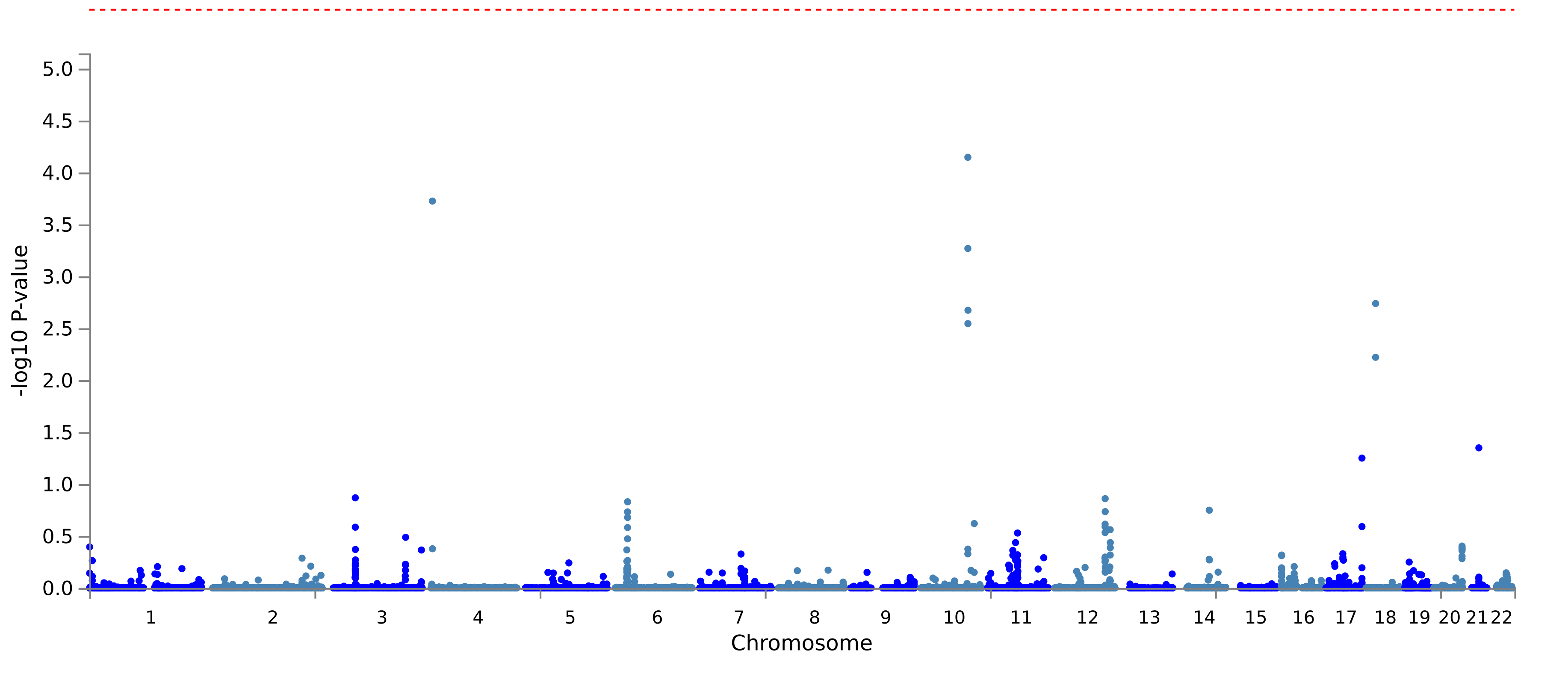


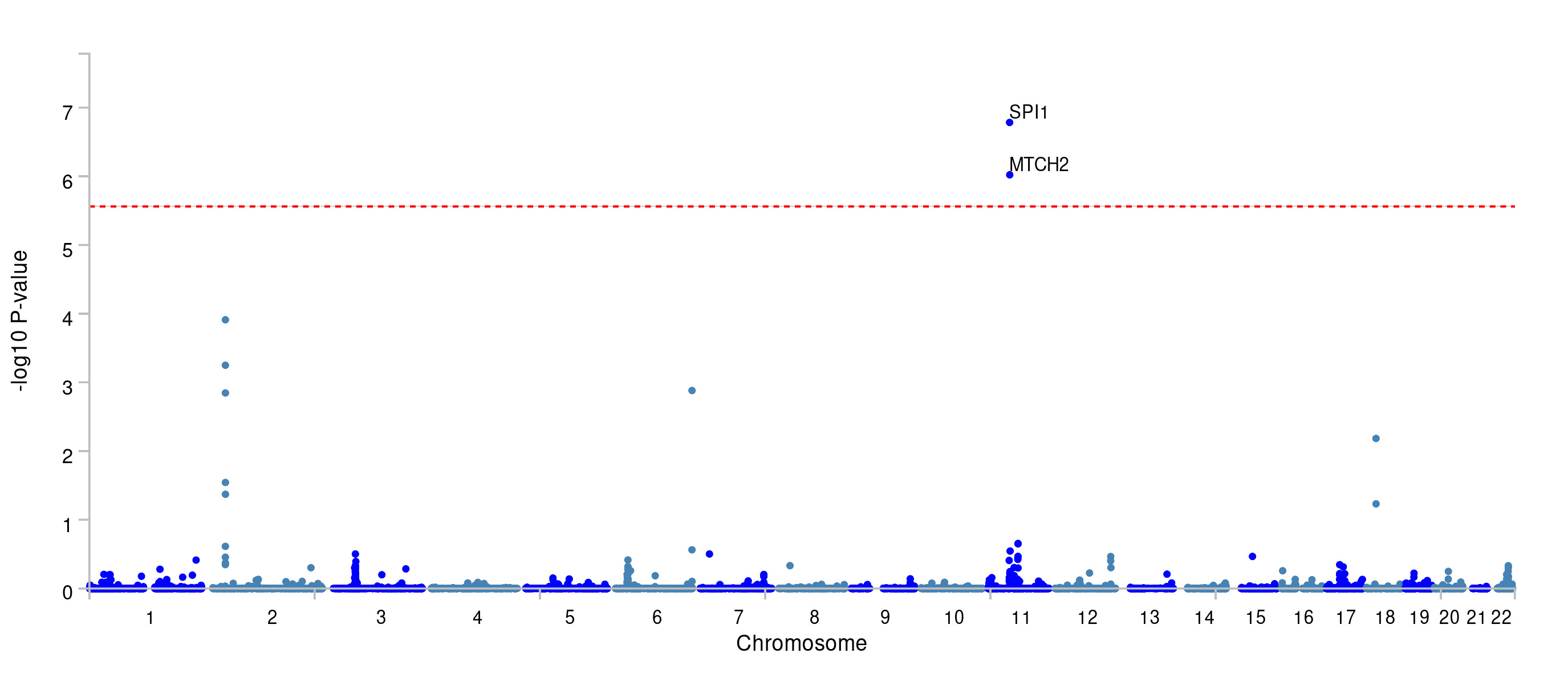


## **Supplementary Figure 10. Gene-based Manhattan plot for cannabis-related traits with the non-cognitive aspects of educational attainment.**

*Note:* The top panel displays results for the joint SNP-level associations of lifetime cannabis use (CanUse) and the non-cognitive components of educational attainment (NonCogEA), while the bottom panel shows results for cannabis use disorder (CUD) and NonCogEA.


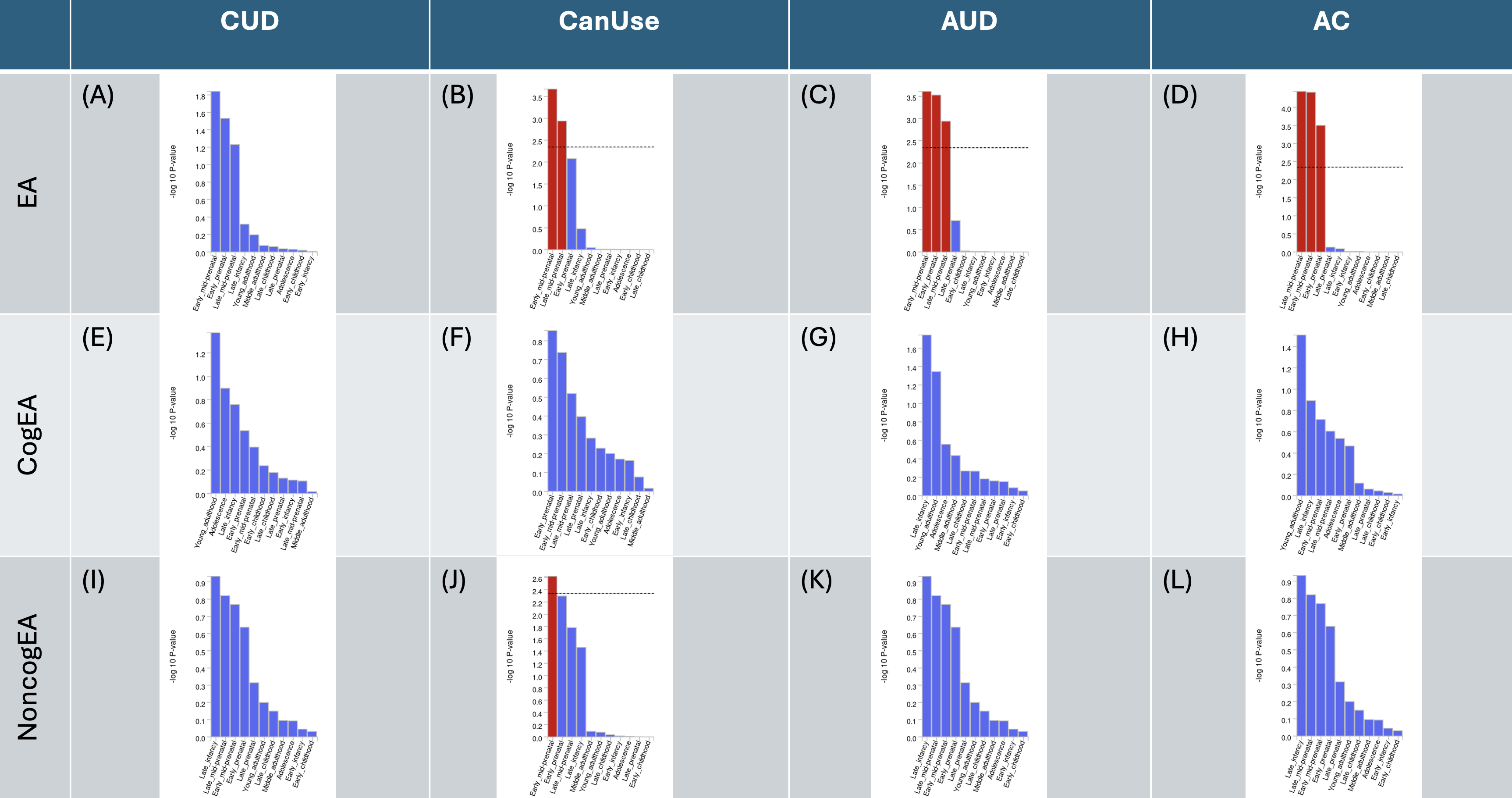


## **Supplementary Figure 11. MAGMA results of BrainSpan 11 different developmental ages for each joint analysis.**

Results that were significant after multiple testing correction are highlighted in red. Panels A-D represent the results of enrichment analysis for joint condFDR analyses of EA with CUD, CanUse, AUD, and AC, respectively. E-H represent those of joint analyses of CogEA with the four substance-related traits, and I-L represent NonCogEA with the four traits.


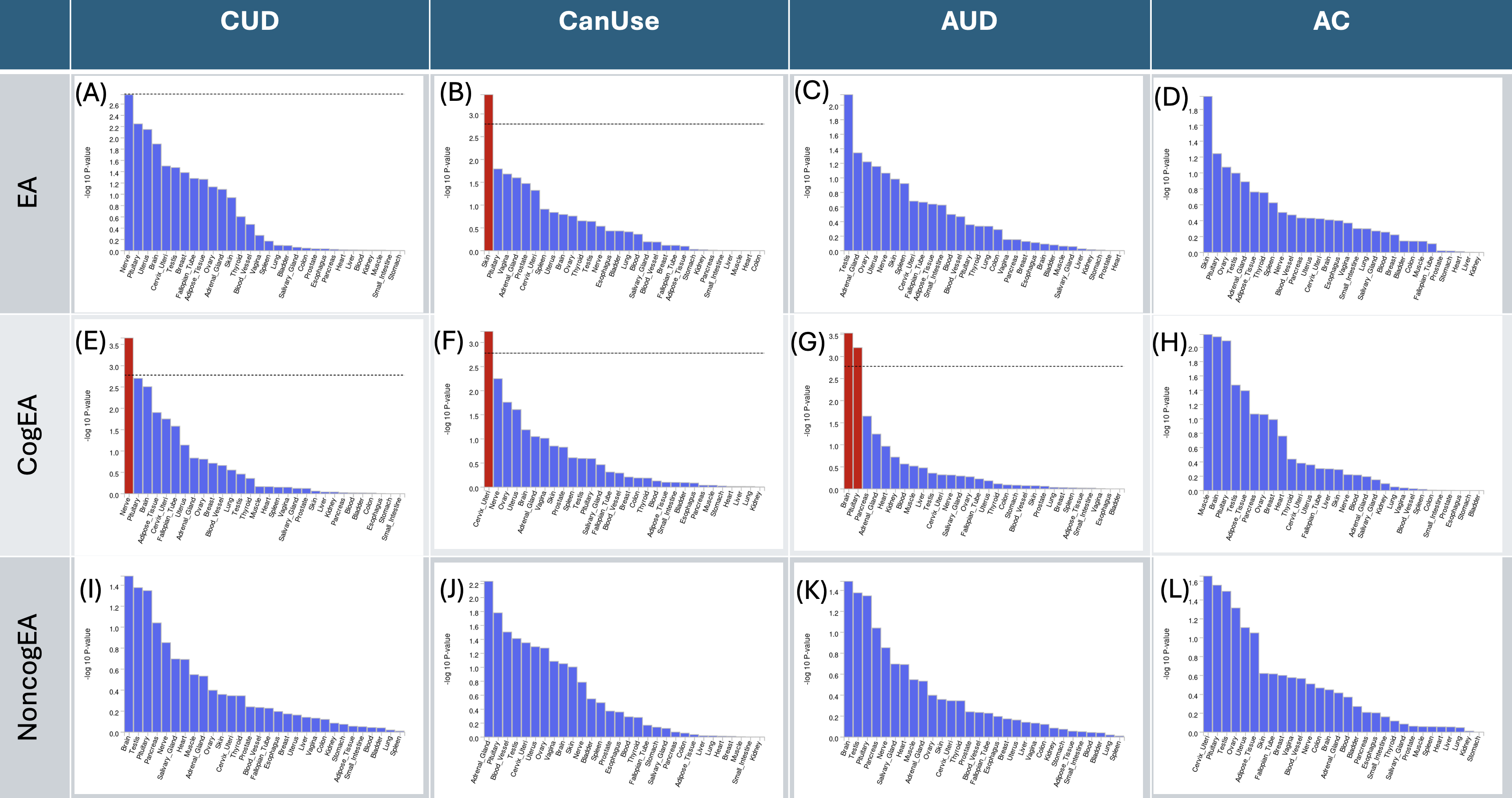


## **Supplementary Figure 12. MAGMA results of GTEx v8 30 tissue types for each joint analysis.**

Results that were significant after multiple testing correction are highlighted in red. Panels A-D represent the results of enrichment analysis for joint condFDR analyses of EA with CUD, CanUse, AUD, and AC, respectively. E-H represent those of joint analyses of CogEA with the four substance-related traits, and I-L represent NonCogEA with the four traits.


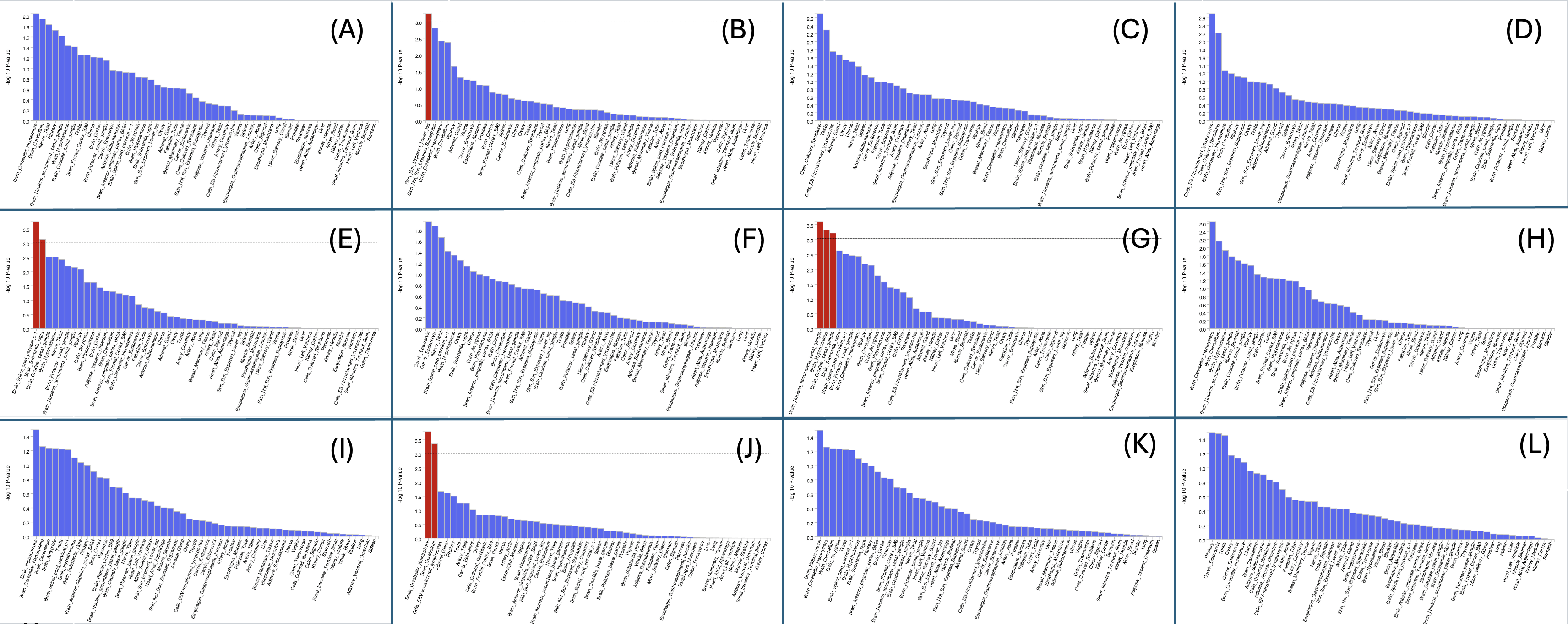


## **Supplementary Figure 13. MAGMA results of GTEx v8 53 tissue types for each joint analysis.**

Results that were significant after multiple testing correction are highlighted in red. Panels A-D represent the results of enrichment analysis for joint condFDR analyses of EA with CUD, CanUse, AUD, and AC, respectively. E-H represent those of joint analyses of CogEA with the four substance-related traits, and I-L represent NonCogEA with the four traits.
